# Analysis of Short-Term Subjective Well-Being/Comfort and Its Correlation to Different EEG Metrics

**DOI:** 10.3390/s26020446

**Published:** 2026-01-09

**Authors:** Betty Wutzl, Kenji Leibnitz, Yuichi Ohsita, Masayuki Murata

**Affiliations:** 1 D3 Center, The University of Osaka, Toyonaka 560-0043, Japan; 2 Graduate School of Information Science and Technology, The University of Osaka, Suita 565-0871, Japan; 3 Center for Information and Neural Networks (CiNet), National Institute of Information and Communications Technology, Suita 565-0871, Japan

**Keywords:** electroencephalography, EEG, subjective well-being, SWB, comfort, thermal comfort, individuality

## Abstract

**Highlights:**

**What are the main findings?**

- Relative power of EEG sensors is related to short-term SWB/comfort.
- The participants could not be grouped into consistent subgroups with similar correlation between their EEG data and their reported short-term SWB/comfort levels.

**What are the implications of the main findings?**

- The reported linear correlation between different EEG relative powers and SWB/comfort also holds when SWB is changed on a 30 s scale and when changed via environmental conditions.
- While our findings are significant, they vary between individual participants, which cannot be grouped into consistent subgroups, at least not with the methods we used.

**Abstract:**

Finding a correlation between physiological measures and subjective well-being (SWB) or comfort has been an active research area in recent years. We focus on short-term SWB measures and their correlation to electroencephalography (EEG) signals in an office environment. We recorded EEG from 30 participants and asked them to report their SWB every 30 s. We analyzed the correlation between the relative power of different frequency bands at various sensor locations and SWB via k-nearest neighbor (k-NN) classification and linear regression. We also analyzed the correlation of the time series themselves at different sensor locations and how they can be classified into different SWB values via k-NN. Then, we tried to cluster participants into subgroups that had a similar correlation between their EEG recordings and their reported SWB. We found that a correlation between relative power and SWB also holds for short terms. However, the results of every single participant of all analyses vary substantially, and we could not find any consistent clustering into subgroups. That implies a huge individuality when it comes to EEG measures and reported short-term SWB.

## 1. Introduction

Feeling comfortable and well has been gaining more attention in recent years. Several different fields, from psychology to economics, focus on making people more comfortable. Diener et al. defined subjective well-being (SWB) as “a broad category of phenomena that includes people’s emotional responses, domain satisfactions, and global judgements of life satisfaction” [1] (p. 277). This term is often used in the field of psychology, where similar terms such as “subjective happiness” [2], “psychological well-being” [3], or “quality of life” [4] have been used. Those measures are closely linked to terms like “comfort”, which was defined by Vink and Halleck as “comfort is seen as a pleasant state or relaxed feeling of a human being in reaction to its environment” [5]. When it comes to HVAC (heating, ventilation, and air conditioning) or building sciences, the ASHRAE standard is often used with its definition of “thermal comfort” as “that condition of mind which expresses satisfaction with the thermal environment” [6].

All these definitions are subjective and are most of the time collected via questionnaires; however, there are also attempts to measure them via physiological signals or at least attempts to find a correlation with them. Especially in psychological studies, measurement tools such as electroencephalography (EEG), functional magnetic resonance imaging (fMRI), or positron emission tomography (PET) are used. While fMRI and PET require extensive machines, EEG is portable and easier to use, so it is often used to evaluate SWB or comfort in office environments.

In our previous studies, we focused on frontal alpha asymmetry (FAA) as our main measure to extract from the EEG. It has already been reported that FAA correlates with SWB in a psychological sense, and it was shown that FAA has a direct correlation to SWB [7,8]. We then reported that such a correlation also holds for short terms (minutes to seconds) and when influencing the participant’s SWB via changing the environment, i.e., temperature and humidity [9]. In a follow-up paper, we discussed that just focusing on the alpha band, as just one frequency band, might not be enough. We analyzed asymmetries between different sensor locations and EEG frequency bands, namely, the delta band (0.5–3 Hz), the theta band (4–7 Hz), the alpha band (8–13 Hz), the beta band (14–30 Hz), and the gamma band (>31 Hz). We concluded that while the asymmetry between the frontal areas in the alpha band is often most strongly correlated to SWB, there are other frequency bands and sensors that should not be neglected [10].

After focusing on asymmetry, we then investigated how other metrics commonly extracted from EEG recordings correlate with SWB. A review by Shi et al. (2024) discusses the application of EEG in thermal environments, focusing on comfort, performance, and sleep quality [11]. Many of the papers discussed in this review use the relative power of the EEG signal to correlate it to comfort, which is, as discussed above, close to the term SWB we used in our previous work. The relative power of the EEG signal, especially in the alpha band and from the right hemisphere, is often correlated with thermal comfort. However, low-frequency EEG in the delta, theta, and alpha bands, as well as high-frequency EEG in the beta and gamma bands, show correlations to thermal comfort. Yao et al. [12] reported on relative power and thermal sensation and describe that the relative alpha power is highest in “neutral” and “slightly cool” states, whereas the relative beta power is highest for “hot”, “warm”, “cool”, and “cold” with an increase in uncomfortable states (“hot”, “cold”). Similar results regarding the relative power of the alpha and beta bands were found in [13]. Son et al. [14] focused on the thermal comfort vote (TCV) and analyzed its correlation to relative power. They found that the changes in relative theta power were statistically correlated at the EEG sensor locations Fz, Cz, and POz. Zhang et al. [15] recorded EEG with a similar Emotiv device as we used and found that the absolute power of the alpha and beta bands at the channels F8, F4, AF4, and T8 was the lowest under neutral conditions. Lower levels of the delta band were found at F4 and F8. In accordance with our previous papers [9,10], different brain regions show different correlations to thermal comfort, with the activity in the frontal lobes showing a stronger correlation with thermal comfort [16,17]. When comparing our experimental setup to the existing literature, we have two main advantages. The first is that we have a finer temporal resolution of SWB recordings. During our experiment, we record SWB every 30 s, while most of the literature records one SWB value per environmental condition. The second advantage of our setup is the use and analysis of 14 EEG channels, which most often exceeds the number of EEG channels used in similar studies. That means our main focus in the first section of this manuscript is to see if results reported in the literature are flexible and can change on time scales from minutes to seconds, or if they need more time to adjust. Also, the larger availability of sensor locations allows us to define brain regions underlying those fluctuations more precisely. Moreover, we explore different metrics and contrast them, as well as aim to categorize the participants into consistent clusters.

In this paper, we will discuss two major research questions (RQ). The first one is about relative power. As it is often reported and discussed, we also want to analyze our dataset and see what correlation we can find, i.e., RQ1: Is the relative power of different frequency bands correlated to short-term SWB?

Then, we will move on to a more general question. We have focused on asymmetrical values in the past, so now we will discuss the correlation of relative power to short-term SWB in more detail. Besides that, we also tried different algorithms. As our results differed hugely between participants and conclusions so far were all drawn on a group level, we tried to classify the participants into different groups for members of one group to have a more similar correlation between their EEG data and SWB than members of different groups. Inter-individual variability reflects underlying biological heterogeneity. EEG captures oscillatory dynamics, connectivity, and excitation–inhibition, which are all neurophysiological traits. Moreover, several papers have been reporting on EEG phenotypes, subject-specific traits, or defining responders vs. non-responders to medication, e.g., [18,19]. While EEG features may vary between individuals, clustering makes results more interpretable and can give approximations of response patterns. While it is not clear a priori that clusters exist, it is worth exploring the possibility. Thus, the second research question, RQ2, is as follows: Are there underlying clusters of participants with a similar correlation between their EEG measures and the reported short-term SWB?

## 2. Materials and Methods

### 2.1. Experiment

**Figure 1:**
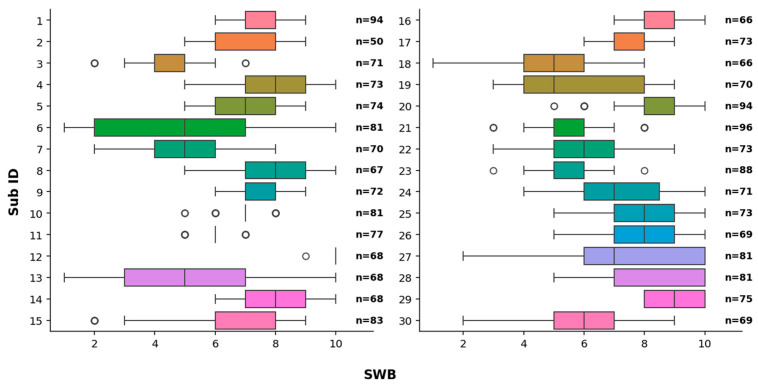
Box plot of SWB values reported by each participant. The x-axis gives the SWB values, and the y-axis gives the participants’ number as Sub ID. The value n in each line stands for the total number of SWB recorded for that specific participant.

Parts of the results of this experiment have already been published in [9,10], where we focused on EEG asymmetry and its correlation to SWB. The experiment was performed in 2022 at the University of Osaka, from which we had received ethical approval for the study. We included 30 students (28 right-handed, 2 left-handed, 16 males, 14 females, ages 22.3 ± 4.2 years) in the study. The handedness was noted as self-reported by the participants. The participants were brought to an experimental room which was equipped with a desk, a chair, several shelves on which we placed temperature–humidity sensors and heating/humidifying devices. The experiment was explained to the participants in either Japanese or English, and then they signed a written consent form. The experimenter then placed an EEG headset (EPOC X, EMOTIV, San Francisco, CA, USA) on the participant sitting in the chair at the desk. One of six different temperature–humidity settings was chosen, and the EEG was recorded for up to 9 min per setting. During these recordings, a SWB value was given verbally by the participant every 30 s. The SWB values were given as integers from 1 to 10, with 10 representing the highest comfort level with the environmental conditions (temperature, humidity) and overall feeling well. A SWB value of 1, on the other hand, represented the lowest comfort level and corresponded to situations where the participants felt like leaving the current environment. After the recording, the participant was told to relax while the experimenter changed the temperature–humidity settings. A more detailed description of the experiment can be found in [9], where the Supplementary Material lists all the temperature–humidity data recorded with sensors developed in [20]. Figure 1 shows the distribution of the SWB reported by each participant as well as the total number of usable SWB recordings. As one can see, the distribution is different between participants, but that does not interfere with our analysis, as in the beginning, we focus on different SWB values of individual participants and then explicitly try to group similar participants.

### 2.2. EEG Preprocessing

The EPOC X EEG headset used in the experiment follows the standard 10–20 electrode placement system with 14 channels (AF3, F7, F3, FC5, T7, P7, O1, O2, P8, T8, FC6, F4, F8, and AF4), the common mode sense (CMS) at P3, and the driven right leg (DRL) at P4. The headset has an internal sampling rate of 2048 Hz, which is downsampled to a rate of either 256 or 128 Hz, which we used later for our experiments. The bandwidth for the recording is 0.16–43 Hz. The device also applies a digital notch filter at 50 and 60 Hz automatically. The EEG preprocessing was then performed in MATLAB (R2022a) [21]. We followed HAPPE (The Harvard Automated Processing Pipeline for Electroencephalography) [22] using EEGLAB (v2022.0) [23], and MARA (Multiple Artifact Rejection Algorithm) [24,25]. The detailed pipeline is described in [9]. We then interpolated the bad channels with EEGLAB using the default spherical interpolation.

### 2.3. Relative Power

We used the last 10 s before the SWB was reported by the participant to calculate the relative power. We chose this interval because extracting metrics from that specific interval yielded the highest correlation to SWB in our previous paper; see [9] for a detailed discussion about interval lengths and temporal locations. Then, the relative power of the delta (0.5–3 Hz), theta (4–7 Hz), alpha (8–13 Hz), beta (14–30 Hz), and gamma (31–55 Hz) bands was calculated at each channel.

#### 2.3.1. k-Nearest Neighbors (k-NN)

**Figure 2:**
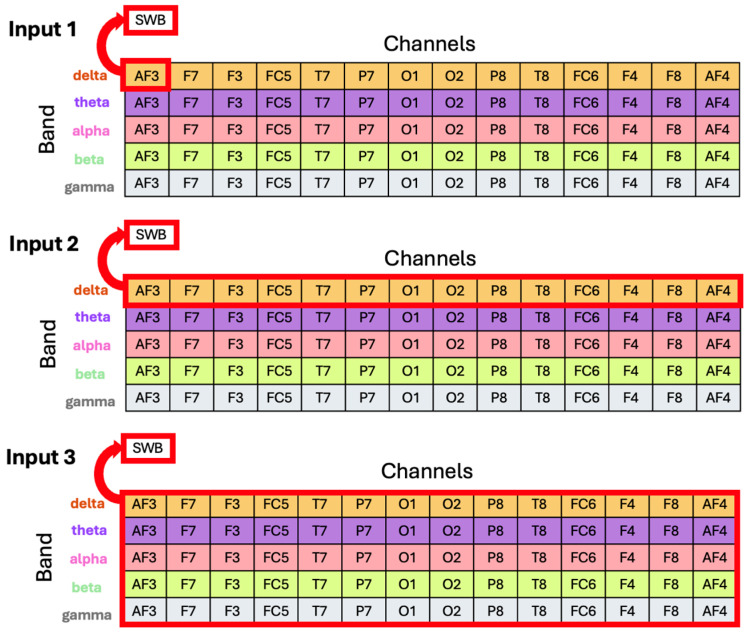
Overview of the three different inputs. Different colors and lines represent different frequency bands. The red rectangles show one exemplary choice for each input.

The first approach we want to discuss is the k-nearest neighbor (k-NN) classification [26]. It is a well-established classification algorithm that labels a new data point based on the labels of the majority of its k-NN. Our dataset is highly imbalanced (see Figure 1), so we decided to use k = 1, i.e., assign the label to that of the closest neighbor. Using more than one neighbor may result in wrong classifications because it is more likely to find points of majority groups near an arbitrary point. If, for example, k = 3 is chosen and the training set contains just one data point for a specific SWB, a new point will not be classified as that SWB. For those reasons, we set k = 1. Although there are different methods to balance a dataset before classification, none of them are suitable or well-established for use with multivariate time series across multiple classes. In the following, we use the same algorithm with a multivariate time series as input. Since we want to be able to compare these sections, we choose not to balance the data beforehand, but rather to set k = 1. We ran the algorithm for each participant individually with three different inputs (see Figure 2):

- Input 1: Relative power of a specific frequency band on a specific channel, i.e., 1 relative power value labeled with 1 SWB value.
- Input 2: Relative powers of a specific frequency band at each channel, i.e., 14 relative power values labeled with 1 SWB value.
- Input 3: Relative powers of all frequency bands at each channel, i.e., 70 relative power values labeled with 1 SWB value.

We deleted all data for an SWB that was just given once. Then, we used a leave-one-out (LOO) [27] approach and calculated the mean squared error (MSE). To evaluate the performance of a classifier, the accuracy score is often used. This metric gives the portions of all classifications that were correctly classified. However, in our case, the MSE might be more useful. The SWB values reported by the participants have a numerical order; hence, misclassifying a SWB value as being close to the actual value (e.g., SWB = 3 misclassified as 4) should be less severe than misclassifying it as a distant value (e.g., SWB = 3 misclassified as 9). We tested the dominant results for statistical significance using a chi-squared goodness-of-fit and a binomial test as implemented by SciPy (1.12.0) [28].

#### 2.3.2. Linear Regression

As mentioned in Section 1, many papers discuss the correlation between SWB (or comfort or thermal comfort) and relative power in the form of a linear regression. In order to compare our results to those papers, we also performed a linear regression. Our dataset is highly imbalanced. Participants would report SWB values of 6, 7, or 8 a lot more often than 1, 2, or 10, as can be seen in Figure 1. Also, the number of different SWB values that were reported varied highly between participants. In order to balance the dataset for each participant, we performed the Synthetic Minority Over-sampling Technique (SMOTE) [29] using its implementation in imbalanced-learn (0.10.1) [30] with Python (3.9.18) [31] and scikit-learn (1.1.3) [32]. This algorithm generates samples similar to the existing ones so that the dataset has an equal number of samples for each group. We performed the same SMOTE algorithm as described in [9], but instead of the FAA values as input, we used the different relative power values. A linear regression was performed using the balanced data as input, using the method described in [32]. SMOTE produces slightly different outputs each time it is run, so we performed each evaluation 10 times and calculated the average slope for each participant individually. To ensure reproducibility, we set a fixed random seed for each run of SMOTE. Specifically, we set the variable SMOTE_seed = j for j = 1, 2, …, 10. Then, a group statistic was determined considering all the slopes from each participant with a two-sided *t*-test to see if the slope was equal to 0. The *p*-value and 95% confidence interval (CI) were determined using [28]. The CIs are for group-level and use the mean slope per participant as the unit of analysis. We performed this linear regression for relative power values from 5 frequency bands and also measured at each of the 14 sensors. Hence, we controlled the false discovery rate (FDR) at q = 0.05 using the Benjamini–Hochberg procedure implemented in [33].

### 2.4. Time Series

In our previous analyses, we focused on pairs of asymmetries (mostly of frontal areas) and SWB values, i.e., (FFA, SWB) [9,10]. In the previous section, we analyzed relative power values and their correlation to SWB. Now, we analyze the time series themselves and do not only represent them by an extracted metric. The EEG headset that we used records time series from the 14 channels for up to nine minutes each session. In order to reduce the data, we decided to again focus on the last 10 s before the times when the SWB was reported by the participant. This leaves us with a time series of 1281 data points each. For each participant, we have around 70 sets of 14 EEG time series with one out of 10 SWB values as labels. As described above, SWB scores in the middle of the range were more frequently reported, which resulted in an imbalanced dataset. Searching the literature, we found that most approaches to artificially balance imbalanced datasets of time series have univariate data (a single time series) and only 2 classes to distinguish. Such an approach is not directly applicable to our setup, as we have multivariate time series from 14 EEG channels and 10 classes of possible SWB values. Hence, we turn again to the nearest neighbors’ (NN) approach [26] which we also used in Section 2.3.1. Such an approach finds the k samples in the dataset that are closest to the point to classify. The only difference from the above is that now the input is a time series or a set of time series, whereas before we focused on single values or sets of single values. Since we still have an imbalanced dataset, we again chose k = 1. We used all 14 channels together as input, but then also filtered the data into different frequencies and used just single channels as input. We again try three different inputs, analogously to Figure 2 and Section 2.3.1:

- Input 1: Time series filtered into a specific frequency band on a specific channel, i.e., 1 time series labeled with 1 SWB value.
- Input 2: Time series filtered into a specific frequency band at each channel, i.e., 14 time series labeled with 1 SWB value.
- Input 3: Time series filtered into all specific frequency bands at all channels, i.e., 70 time series labeled with 1 SWB value.

Analogous to the above, we use the MSE between the real and predicted SWB values to evaluate if the result of a specific classifier fits our purpose better than using just accuracy. We performed an LOO approach for each participant and then calculated the MSE between all left-out SWB values and the true SWB values. We used the implemented function in sktime [34] and also used different distance metrics for the k-NN algorithm, namely, Euclidean and Dynamic Time Warping (DTW). We tried both methods, and while DTW, which is the default method in sktime, took longer to compute, the numerical results were not much better. Therefore, we decided to use the Euclidean metric as a distance.

### 2.5. Clustering

We used different methods to study the connection between EEG recordings and SWB. The results of the analyses in the last two sections are similar to the ones we found when we used asymmetrical values, namely that effects were mostly just observed at the group level, with big differences noted among individual participants. Now, we explore the possibility of finding subgroups of people who have similar relationships between a specific EEG measure and their reported SWB. We look at how these relationships are the same within each subgroup and different between the subgroups. In order to apply a clustering algorithm, we need to have a distance metric. Two participants can be considered as having a similar relationship between an EEG measure and SWB if the specific parameters for an algorithm determined for one participant of a subgroup also work well for other participants in the same subgroup and not so well for participants of different subgroups. Thus, we identify the most suitable parameters for an algorithm to describe SWB utilizing Participant A’s EEG data as the input. Then, we use the algorithm with the identified parameters to predict Participant B’s well-being. How well Participant A’s algorithm works for Participant B shows how close they are. When choosing a metric to determine how well SWB values from Participant B were determined, we must consider the diversity in SWB values given. Different participants report different numbers of SWB values, so using an absolute measure would not be representative and would distort the metric when comparing all the different participants. Assume that Participant A reports SWB values from 5 to 7, whereas Participant B reports values from 2 to 9. The maximum absolute error for Participant A’s prediction model is 2, whereas Participant B’s is 7. This shows that a relative metric is more suitable than an absolute one. We decided on a modified version of the mean absolute error in which we calculate the distance matrix between Participant A and Participant B using SWB_true_ and SWB_pred_ of Participant B via the following formula:

(1) $${dist}_{AB}\left(SWB_{true},SWB_{pred}\right)=\frac{1}{n}\frac{\sum_{i=1}^{n}\left|SWB_{true,i}-SWB_{pred,i}\right|}{unique(SWB_{true})}$$

Here, SWB_true_ are the recorded SWB values from Participant B, SWB_pred_ are the predicted SWB values using Participant B’s EEG data on an algorithm optimized for Participant A, n is the total number of SWB values recorded from Participant B, and the function unique gives the number of unique values of the dataset.

By dividing by the unique number of SWB values, we account for the possible different maximum absolute errors for different participants, and this lets us compare $dist_{AB}$ between all recorded participants, no matter how many data points are available for each individual participant.

For the clustering, we want to focus on three relationships between EEG data and SWB, namely, the correlation between SWB and the following:

1. Frontal alpha asymmetry (FAA) (see [9]);
2. Relative power (see Section 2.3);
3. EEG time series (see Section 2.4).

The FAA uses the results published in [9]. In this paper, we focused on the FAA and its correlation to short-term SWB. The experiment was the same as discussed in this work. After standard EEG preprocessing, we filtered the EEG time series of each participant into the alpha band and calculated the asymmetry at the two most frontal sensors, i.e., AF3 and AF4, via the following:

(2) $$FAA=mean\left(log{\;pow}_{R}-log{\;pow}_{L}\right).$$

We then performed a group analysis to see if the average slope of the linear regression (FAA, SWB) was greater than zero. We analyzed different intervals over which the FAA was calculated and found that the FAA calculated over the last 10 s prior to reporting the SWB resulted in the most statistically significant correlation.

Using the analyses performed in that paper, we found a linear correlation (FAA, SWB) for each individual Participant A, resulting in individual slopes $k_{A}$ and intercepts $d_{A}$. These individual parameters can now be used for the other participants. The distance between Participant A and Participant B can be calculated using the formula above (1), with $SWB_{true}$ being the recorded SWB values of Participant B and $SWB_{pred}$ being the SWB calculated from the input data of Participant B using the parameters from Participant A for the linear model, i.e., slope $k_{A}$ and intercept $d_{A}$.

The second algorithm uses relative power as input. The relative power of each of the five frequency bands was calculated at each of the 14 sensors. We then used a k-NN approach to classify the relative power values to the SWB values. The frequency band and sensor that produced the best result, i.e., lowest MSE, were saved for each participant. Now, we can compute the distance between Participant A and Participant B via (1), with $SWB_{true}$ being the recorded SWB values of Participant B and $SWB_{pred}$ the SWB calculated from the data of Participant B using a k-NN approach trained on the data of Participant A and using the frequency band and sensor that yielded the best results for Participant A. For the linear regression part, we follow the approach described for FAA, but with the slope and intercept of the relative power of the sensor with the lowest *p*-value on the group level.

The third algorithm operates similarly to the second, but it uses time series data instead of relative power values. The time series is filtered into one of the five frequency bands. The filtered time series from a specific sensor serves as input for the k-NN (k = 1) approach. Analogously, the algorithms are performed for all five frequency bands and 14 channels as inputs. Then, the MSE is calculated using LOO for each individual participant. This allows the identification of the best frequency band and sensor for each individual participant. The distance between Participant A and Participant B is calculated by ${dist}_{AB}\left(SWB_{true},SWB_{pred}\right)$, where $SWB_{true}$ represents the recorded SWB values of Participant B, and $SWB_{pred}$ is the SWB calculated from Participant B’s data using the k-NN algorithm trained on the data of Participant A using the frequency band and sensor that yielded the best results for Participant A.

We now have distance matrices from three different approaches. For clustering, we turned to Isometric Mapping (Isomap) embedding [35], which is said to be one of the first approaches to manifold learning. Isomap can be seen as an extension of Multi-Dimensional Scaling (MDS) or Kernel Principal Component Analysis (PCA). Isomap aims to achieve a lower-dimensional embedding while maintaining the geodesic distance between the points. We used Isomap as implemented in sklearn with the size of neighbor set as 1 and a precomputed distance matrix. This matrix excluded participants 12 and 29 as they had too little data. Then, distance matrices of the three algorithms were symmetrized by using the minimum of the distance of Participant A to Participant B and the distance of Participant B to A as follows:

(3) $$dist\left(Sub_{A},Sub_{B}\right)=dist\left(Sub_{B},Sub_{A}\right)=\min(dist\left(Sub_{A},Sub_{B}\right),dist\left(Sub_{B},Sub_{A}\right)).$$

We applied the embedding for each algorithm and obtained labels for our participants when separating the participants into either 2, 3, 4, 5, or 6 groups via k-means, also as implemented in sklearn. K-means is a clustering algorithm that finds k-groups by repeatedly assigning a sample to the nearest cluster center. In order to determine the number of clusters that yield the best result, we computed the Silhouette coefficient [36] with its implementation in sklearn. This score gives the mean Silhouette coefficient over all samples, where a single Silhouette coefficient is given as follows:

(4) $$s=\frac{b-a}{max(a,b)}.$$

Here, a is the mean distance between the sample and all points of the same class, and b is the mean distance between the sample and all points of the nearest cluster. The score is a number between −1 and 1 with higher values indicating denser and well-separated clusters.

**Figure 3:**
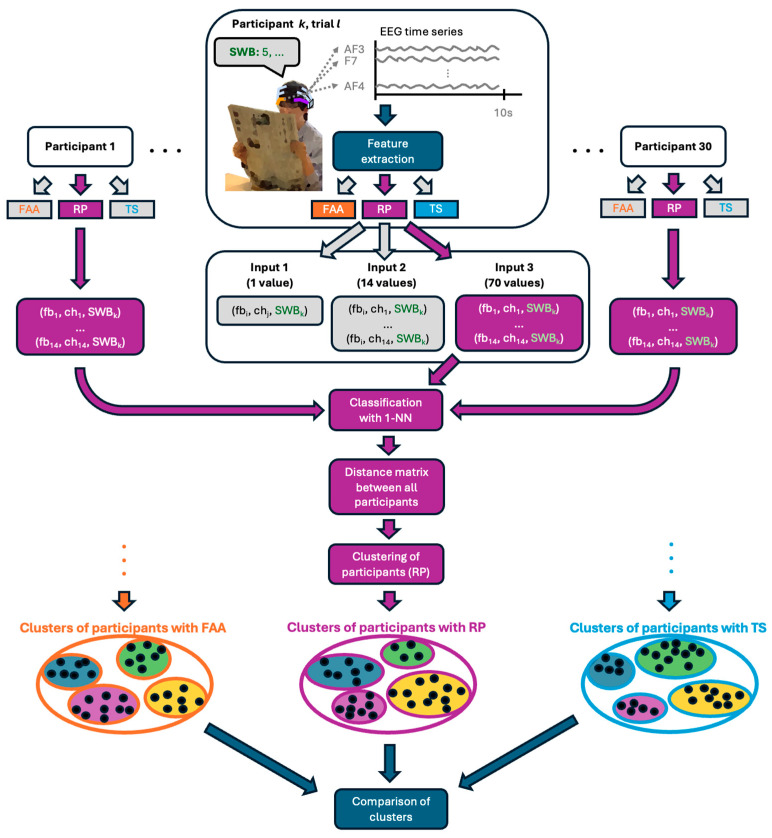
Schematic overview of the processing pipeline for comparing clusters of participants derived from different metrics of the EEG data. We consider the correlation of the SWB values with frontal alpha asymmetry (FAA), relative power (RP), and the EEG time series (TS). We use three different combinations that map the frequency band (fb) and channel (ch) to SWB values, which are then classified with k-NN (k = 1), and from which a distance matrix is obtained that is used for clustering the participants. Finally, the outcomes of the clusterings can be compared with each other. Note that the clusters derived from the linear regression of RP is discussed in the manuscript but not represented in this figure.

Now we have clustered the participants into subgroups according to the relationship between their EEG recordings and SWB. This relationship was determined using three different approaches, using either (1) FAA, (2) relative power of different frequency bands at different sensors, as well as linear regression, or (3) EEG time series of different frequency bands at different sensors as input for the algorithm. The next step is to determine if the partitions differ between the algorithms or persist across them. Figure 3 shows an overview of the entire process. One commonly used metric to determine the consistency of clustering is the so-called Rand index, which measures the similarity between two clusterings by comparing how many pairs of elements are grouped together or separately in both partitions, divided by the total number of possible pairs. The adjusted Rand index gives a similar measure but adjusts for a random classification of all participants [37,38]. We used the adjusted Rand score as implemented in sklearn and compared the partitions pairwise. If the partitions remain consistent, it suggests that the clusters reflect an underlying similarity within a subgroup of participants in how their EEG recordings predict individual SWB in response to environmental changes.

## 3. Results

### 3.1. Relative Power

#### 3.1.1. Relative Power and k-NN

Since most participants reported a different number of SWB values, we cannot directly compare the absolute values of the MSE between participants; however, we can see which combinations of frequency bands and channels yield the lowest MSE for each participant. We had to exclude Participant 12 from the whole analysis since this participant always reported the same SWB value.

##### Relative Power and k-NN: Input 1

As the first input, we used the relative power of a specific frequency band on a specific channel individually. Thus, we run the algorithm for each of the 14 channels and five relative frequencies individually, labeled with one SWB value. Comparing the results, no consistency was found between which channels or frequency bands resulted in the lowest MSE for each individual participant. Before moving on to the results, we want to point out one more time that different participants reported different numbers of SWB values. Hence, we cannot compare the calculated MSE between participants directly. If one participant reported 10 different SWB values, the MSE was higher than that of a participant who reported two neighboring SWB values. Starting with the relative power of the delta band, we find that low MSEs are reached for eight participants in the parietal regions, five participants at P7, and three participants at P8. It seems that when it comes to SWB or comfort, data from the sensors on the left hemisphere have a slightly stronger correlation to the delta relative power than data from the sensors on the right hemisphere. However, those are just tendencies and do not hold for all participants. The algorithm for the two left-handed participants resulted in the lowest MSE for the relative delta power of frontal sensors, but also, those two sensors are located on opposite hemispheres. The relative power of the theta band also results in the lowest MSE for 19 participants for sensors on the left hemisphere, with P7 giving the best results for 5 participants, but just two of them have also P7 as the best sensor for the relative delta power. Contrary to the delta band, this time, also an occipital sensor, namely O1, yields low MSE for four participants. The sensors for the two left-handed participants are different; however, they are both located on the left hemisphere. The relative power in the alpha band results in the lowest MSE for nearly half of all participants, i.e., 15 participants, for sensors on the left hemisphere. The sensor that gives the lowest MSE most often is the occipital sensor O2, namely, for four right-handed and one left-handed participant. Next, the relative beta power shows a lower MSE for sensors on the left hemisphere for 19 participants. The sensor resulting most often in the lowest MSE is F4 for four right-handed and one left-handed participants. The last relative power analysis was the relative power of the gamma band. Here, we find slightly more often sensors on the left hemisphere yielding lower MSE, with the most frequent ones being AF3 and T7, for four right-handed participants. The two left-handed participants show the lowest MSE for sensors on the right hemisphere, namely for sensors FC6 and F8.

**Figure 4:**
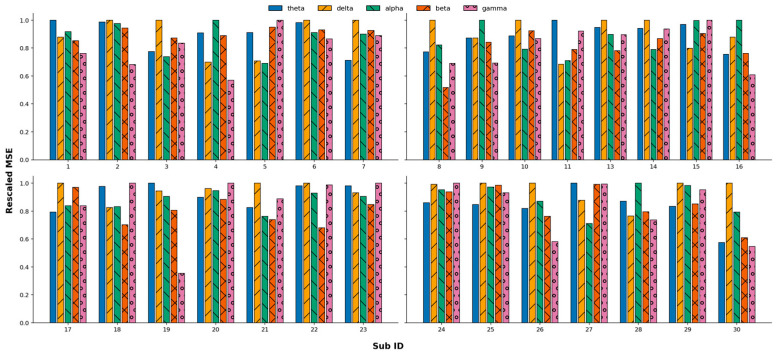
Comparing the lowest MSE of each participant over different frequency bands. The x-axis gives the Sub ID, the y-axis the rescaled MSE. Each participant’s MSE was divided by the largest of the five MSEs. Different colors and patterns represent different frequency bands. The participants with Sub ID 10 and 22 are left-handed; the rest are right-handed.

The overall lowest MSE is found for the relative power in the delta band for five participants, for two participants at P7, for two participants at F7, and for one participant at AF4. The relative power in the theta band shows the lowest MSE for two participants (at FC6 and P7). The relative power from the alpha band results in the lowest MSE for four participants, twice at F3 (with one being a left-handed participant) and at P8 and O2 for one participant. The relative power of the beta band yields the best overall results for seven participants, namely at AF4 for two participants and O2, T7, T8, FC6 (left-handed), and F4 each for one participant. The relative power in the gamma band results most often in the lowest MSE, namely for 11 participants. The specific channels were, three times AF3, twice F7, and F4, T8, O2, P7, FC5, and T7 each once. Full tables listing all MSE for each relative power on each channel can be found in the Supplementary Section (Supplementary Tables S1–S5). Figure 4 below gives an overview of the MSE of each frequency band and each participant. The lowest MSE from each frequency band was chosen for each participant and, for comparison, divided by the largest one of the five frequency bands. That means the highest bar for each participant shows a rescaled MSE = 1 and stands for the frequency band that performed worse for that specific participant. The lowest bar on the hand stands for the frequency band that gives the lowest MSE. This normalization was performed because of the variety of SWB values between participants. The MSE of a participant with a high range of SWB values is naturally higher than for a participant with a low range of SWB values. Normalization now shows the reference to the biggest MSE, and thus, individual participants’ results can be compared. Different colors and patterns represent different frequency bands.

##### Relative Power and k-NN: Input 2

This input groups channels together. The inputs were the relative powers of a specific frequency band at each channel, i.e., 14 relative power values labeled with one SWB value. Table 1 gives an overview of the lowest MSE values for each participant and the corresponding frequency band. A detailed table containing all specific MSE values for each participant and band can be found in Supplementary Table S6. The data from the delta band gives the best results for only 2 participants (one left-handed), the relative performance of the theta band for 3 participants, the alpha band also for 3 participants, the beta band for 5 participants, and the gamma band for 16 participants (one left-handed). Although there are differences between participants, it appears that the relative gamma power dominates in this analysis and has the lowest MSE for most of the participants. We performed a chi-squared goodness-of-fit test and found that the distribution differed significantly from chance (χ^2^(4) = 23.241, *p* = 0.0001). The gamma band led to the best results significantly more often (standardized residual r = 4.24). The binomial test confirmed that the gamma band result exceeded a level expected by chance, i.e., *p* < 0.0001.

##### Relative Power and k-NN: Input 3

After performing the classification using the relative power of all frequency bands at all channels, i.e., 70 relative power values labeled with one SWB value, we can compare the results of all three inputs for each participant, see Table 2. Using Input 1 yields the lowest MSE for six participants, with three of them using the relative beta power on a specific sensor as input; however, those sensors are different for all three participants. Input 2 shows the lowest MSE for 14 participants, with the majority showing that the relative gamma power leads to the lowest MSE, i.e., 10 (including one left-handed) out of 14 participants. Input 3 results in the lowest MSE for eight participants (including one left-handed). We see a high diversity of which input leads to the lowest MSE when comparing participants; however, using Input 2 with the relative gamma power yields the lowest MSE for many participants.

#### 3.1.2. Linear Regression of Relative Power and Short-Term SWB

In this section, we focus on the linear relationship between relative power and SWB. The significant results (*p*-value < 0.05, uncorrected) of the two-sided *t*-test are shown in Table 3; a full table including results with *p*-values > 0.05 can be found in the Supplementary Material (Supplementary Table S8). Several tests showed uncorrected significance, but none of those survived the FDR correction at q = 0.05. All the results exclude Participant 12, who just reported the same SWB throughout the experiment, and the two left-handed participants, resulting in a degree of freedom of 26. The mean slope of the linear regression of the relative power of lower frequency bands, i.e., delta and alpha, is negative, whereas it is positive for higher frequency bands, i.e., beta and gamma. When focusing on the channels, we can see that only channels in the frontal and temporal regions are listed in Table 3. For the delta band, we find two frontal channels (AF3, F7) that have an uncorrected significant negative correlation to SWB, whereas for the alpha band, there are two temporal sensors (T7, T8). The relative beta power has an uncorrected significant positive linear correlation when measured on two frontal channels (F7, FC5) and the relative gamma power with three frontal channels (AF3, F7, FC5), as well as one temporal sensor (T7). It is also worth mentioning that all of those sensors, except T8, are located on the left hemisphere. The mean slope for the left-handed participants for the combinations listed in Table 3 tends to show the opposite direction, i.e., when the table shows a positive linear relation, the left-handed participants tend to show a negative relation and vice versa. The linear regression of the relative alpha power at T7 resulted in the lowest uncorrected *p*-value and will be used for the clustering in Section 3.3.

### 3.2. Time Series

Now we turn to the results of the time series analyses. Here, we used the entire time series instead of a single value as input, i.e., this section is analogous to 3.1.1 but uses time series instead of relative power values. Again, Participant 12 was excluded due to a lack of variability in the SWB values.

Time Series and k-NN: Input 1

In this section, we describe the results of using the time series of each channel filtered into different frequency bands as input for the k-NN classifier. The full table of the results for filtering the time series into the delta band can be seen in Supplementary Table S9. When focusing on the channels that result in the lowest MSE for each participant, we see that for around half of the participants, that specific sensor is located on the right hemisphere, and for the other half, it is on the left hemisphere. On the left hemisphere, the time series filtered in the delta band results in the lowest MSE for four participants at channel T7 and for three participants at P7. On the right hemisphere, the time series filtered in the delta band at frontal sensors yield lowest MSE, i.e., at F8 for four participants and at P8 and AF4 for three participants each. The analysis of the two left-handed participants yields the lowest MSE for the time series filtered into the delta band at the same sensor, namely F4. The results, when filtering the time series into the theta band, can be found in Supplementary Table S10. Again, the lowest MSE values are found for sensors at both hemispheres. For the left hemisphere, T7 yields the lowest MSE for four participants, and on the right hemisphere, AF4 yields the lowest MSE for five participants. When comparing the four participants whose lowest MSEs are found when using the time series filtered into the delta band at T7 to the four participants with the lowest MSE when filtering into the theta band at T7, we see that they differ from each other, except for one. On the other hand, the three participants whose analyses show the lowest MSE at AF4 when filtering the time series into the delta band also result in the lowest MSE at AF4 when filtering into the theta band. The same is true for the participants whose analyses showed the lowest MSE at P8 when filtering the time series into the delta or theta band. The next frequency band is the alpha band. A full table of those results can be found in Supplementary Table S11. When using the time series filtered into the alpha band, more of the sensors having the lowest MSE are located on the right hemisphere, namely for 16 of the 29 participants. Here, analyses for P8 show the lowest MSE for four participants, also F8 (with one of them being a left-handed participant) and AF4 yield the lowest MSE for three participants. Of the four participants with the lowest MSE at P8, two also show the lowest MSE at P8 when filtering in the delta and theta bands. For AF4, only one participant’s analyses show the lowest MSE at that sensor when filtering in the delta, theta, and alpha bands. Next are the results of the beta band, which are shown in Supplementary Table S12. We again find that slightly more of the sensors resulting in the lowest MSE are located on the right hemisphere. P8 gives the lowest MSE for five participants, and T8 and AF4 for three participants each. On the left hemisphere, T7 gives the lowest MSE for four participants and P7 for three participants. One participant who shows the lowest MSE at P8 when filtering into the delta, theta, and alpha bands also shows the lowest MSE at P8 for the beta band. The last band we filter into is the gamma band (see Supplemental Table S13). Again, 13 of the sensors yielding the lowest MSE are located on the left hemisphere and the rest on the right hemisphere. On the left hemisphere, T7 yields the lowest results for four participants, as well as O1 (with one left-handed participant). On the right hemisphere, F4 yields the lowest MSE for five participants (including one left-handed participant), and AF3 yields the lowest MSE for three participants. In this section, we perform an additional analysis, i.e., not filtering into any frequency band (full data in Supplementary Table S14). In this table, we see that, in 19 cases, the sensor giving the lowest MSE is located on the right hemisphere, with P8 yielding the lowest MSE for six participants, followed by F8 for four participants, and O2 for three participants.

**Figure 5:**
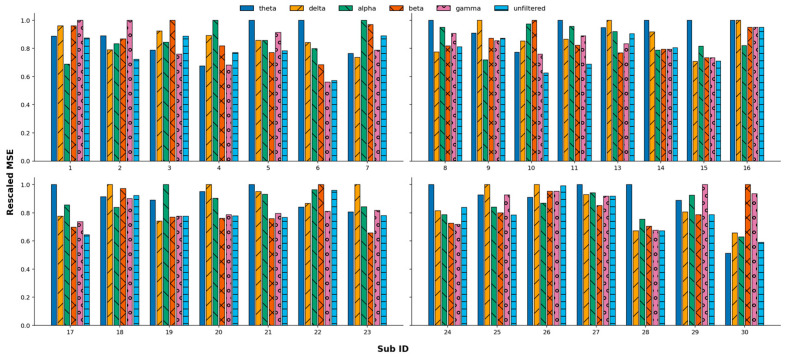
Comparing the lowest MSE of each participant over different frequency bands or unfiltered input. The x-axis gives the Sub ID, and the y-axis gives the rescaled MSE. Each participant’s MSE was divided by the largest of the six MSE values. Different colors and patterns represent different frequency bands. The participants with Sub IDs 10 and 22 are left-handed; the rest are right-handed.

Comparing the results between all frequency bands, as well as for the unfiltered time series as input, we see that filtering the time series into the delta band results in the lowest MSE for two participants, for two frontal sensors, namely AF4 and F7. Filtering in the theta band gives the lowest MSE for five participants, but at very diverse sensors, namely, P8, T8, O2, and T7. The time series filtered into the alpha band shows the lowest MSE for six participants at the sensors FC6, AF3, F8, F3, O1, and P7. Filtering into the beta band gives the best results for six participants for the channels F3, P8, AF3, F4, AF4, and twice at P7. The gamma band analyses show the lowest MSE for four participants, with the channels T8, T7, O1, and F4. Not filtering the time series before the k-NN results in the lowest MSE for seven participants with the channels FC6, F4, FC5/T7 (same MSE for both), F8, AF4, and twice P8. If there is more than one channel giving the lowest MSE, we did not mention it because we cannot determine a “best” channel in those cases. Figure 5 gives a similar overview to Figure 4. It shows the lowest MSE resulting when filtering the time series into a specific frequency band or not filtering the time series. All MSEs of one participant were divided by the largest of them to rescale the MSE values and allow us to compare them across participants.

Time Series and k-NN: Input 2

In this second input case, we group all channels together, i.e., the time series are filtered into a specific frequency band, and then 14 filtered frequency bands (one at each channel) are labeled with one SWB value and used as input for the k-NN algorithm. Table 4 below shows an overview of the results, i.e., filtering in which band results in the lowest MSE and the corresponding MSE for each participant. A table containing the results of each frequency can be found in the Supplementary Material (Supplementary Table S15). The lowest MSE is observed when filtering into the delta band for four participants; into the theta band for three participants, including one left-handed; into the alpha band for seven participants; into the beta band for three participants; into the gamma band for five participants, including one left-handed; and not filtering at all for six participants. Like before, we can see a high variety between participants, and which bands lead to the lowest MSE. We performed a chi-squared goodness-of-fit and found that the distribution does not differ from chance (χ^2^(5) = 2.857, *p* = 0.722).

Time Series and k-NN: Input 3

Comparing Input 3 to Inputs 1 and 2, we find that using the time series filtered into all five frequency bands at all 14 sensors as input results in the highest MSE. For all participants except Participant 4, Input 1 gives the lowest MSE. The full results are shown in Supplementary Tables S16 and S17.

### 3.3. Clustering

So far, we have analyzed all the data. Now we focus on clustering the participants into subgroups according to the relationship between their EEG recordings and SWB. This relationship was determined using three different approaches, namely using either (1) FAA, (2) relative power of different frequency bands at different sensors, or (3) EEG time series of different frequency bands at different sensors as input for the algorithm.

**Figure 6:**
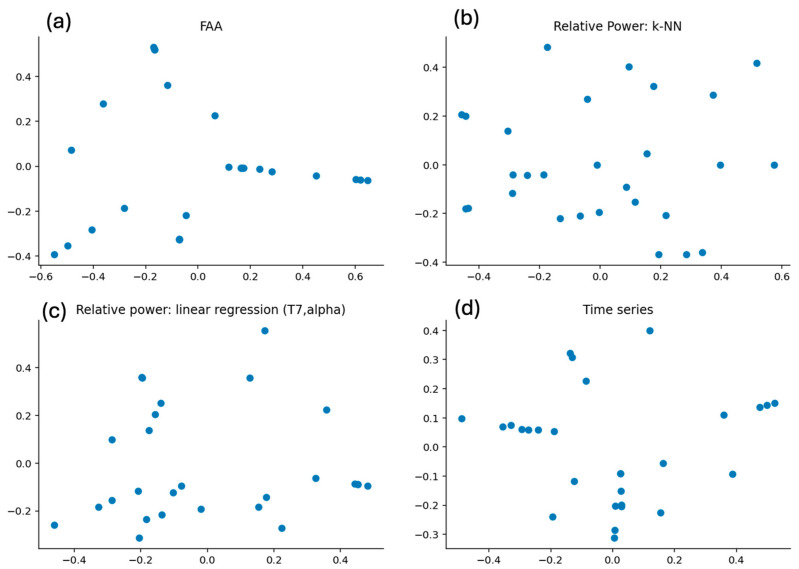
Isomap output of the distance matrix determined via the different approaches: (**a**) FAA, (**b**) relative power using k-NN, (**c**) linear regression of the relative alpha power at T7, and (**d**) time series. Blue dots represent different participants.

The following three figures show the output of the Isomap embedding using the different distance matrices. Figure 6a shows the graphical representation of an Isomap embedding applied to the distance matrix of the FAA. One might guess that those dots can be categorized into four groups. Figure 6b shows that the representation of the embedding of the distance matrix of the relative power makes it hard to suggest any clustering. Figure 6c, on the other hand, when using linear regression of the relative power of the alpha band on channel T7, might suggest a clustering into four groups, similar to Figure 6d, which represents the embedding of the time series distance. This visual inspection implies that there might be some clusters; however, these might be different between the algorithms.

These embeddings serve as the basis for the clustering. We cluster the participants into two, three, four, five, or six groups. The specific assignment of each participant can be found in the Supplementary Material (Supplementary Tables S18–S21). Using the clustering algorithm with the FAA, we find that the Silhouette coefficient is highest for six clusters, namely 0.666, and lowest for two clusters, namely 0.429. Excluding the left-handed participants does not improve those scores, except for k = 3. The two left-handed participants only share the same cluster for k = 2. For the clustering using the distance metric of the k-NN relative power analysis as input, we find the highest Silhouette coefficient for k = 4 at 0.423 and the lowest for k = 2 at 0.354. Comparing those results with the scores from the FAA algorithm, we see that the Silhouette coefficients are lower, which is consistent with the visual inspection of Figure 6a,b, where it seems easier to cluster the embedding of FAA. Excluding the results of the left-handed participants for the clustering using the k-NN distance of the relative power raises the Silhouette coefficient for all tested k, with the highest being 0.553 for k = 3. The left-handed participants are not assigned to the same cluster, no matter the number of clusters. When using the linear regression approach of the relative alpha power at T7 for the clustering, the highest Silhouette coefficient is reached for k = 4 at 0.562, and the lowest for k = 2 at 0.441. The Silhouette coefficients are higher for all numbers of clusters when comparing them to the k-NN approach. Leaving the left-handed participants out improves the Silhouette coefficient only for k = 2, which is also the only partition where the left-handed participants are in the same cluster. The Silhouette Coefficients of clustering using the distance metric determined from the time series algorithm are again higher than for the relative power using k-NN, which is also consistent with what we concluded from the visual inspection comparing Figure 6b,d. The highest Silhouette coefficient for the clustering using the time series algorithm is reached for k = 4 with 0.614, and the lowest for k = 6 with 0.43. Repeating the same analysis without the left-handed participants increases the Silhouette coefficient for k = 2, 5, and 6, but not for k = 3 and 4.

The next step is to determine if the partitions differ between the algorithms or persist across them. The pairwise calculated adjusted Rand indices are given in the following Table 5.

The best alignment is found when comparing the partitions resulting from the FAA and the time series algorithm, with the highest alignment for k = 2, with 0.603, which means that about 60% of pairwise comparisons match after adjusting for chance. Comparing the clusters that result from the relative power using k-NN to either clusters resulting from the FAA, the linear regression of the relative alpha power at T7, or the time series algorithm does not yield alignment. The partition resulting from the FAA algorithm and the linear regression of the relative alpha power at T7 also yield some alignment with the closest alignment for k = 4, with about 40% of pairwise comparisons matching after adjusting for chance.

## 4. Discussion

### 4.1. Relative Power

#### 4.1.1. Relative Power and k-NN

When using Input 1, namely, individual relative power values at different sensors, we find a high variety in which the combination of sensor and frequency band yields the lowest MSE for individual participants. We want to point out once more that we cannot compare MSE between participants, as different participants have reported different numbers and ranges of SWB values. The individually reported SWB values serve as the labels for the input. If Participant A just reported SWB values 3, 4, and 5, the possible maximal MSE is intrinsically smaller than for Participant B, who reported all values from 1 to 10. Keeping that in mind, we can focus on comparing the results of one participant using different relative powers at different sensors as input. That means that we found the best combination of sensor and relative power for each participant individually. If several combinations yield the lowest MSE, we excluded them from the discussion as no clear “best” combination could be determined. While the results show high diversity between participants, we can say that higher frequency bands, i.e., beta and gamma, tend to be chosen more often as the best combination. That indicates that while in the literature several frequency bands, also lower ones like the delta and theta bands, are discussed to show statistical correlation to SWB, we argue that the relative powers of higher frequency bands are more closely related to short-term SWB. When focusing on SWB that changes quickly, it seems that the relative beta and gamma power give the most reliable representations. When focusing on the specific sensor, where the relative power is calculated, giving the lowest MSE, it varies a lot for individual participants, as they span from frontal regions to occipital regions. It is also worth mentioning that there is no consistency in the results of our two left-handed participants. As their number is very low, we cannot say whether this is representative of all left-handed people or just the case for the two left-handed participants in this study.

Moving on to the second input, where we group sensors together, but analyze the relative powers of each frequency band separately, we find that for the majority of participants, the analyses of the relative gamma power yield the lowest MSE when comparing the results of different frequency bands. This is not surprising considering that the relative power of the gamma band at a specific channel often yields the lowest MSE for Input 1, but the specific sensor that yields this lowest MSE varies. When comparing the MSE from the participants with the lowest MSE found for the relative gamma power at any sensor for Input 1 to Input 2, we see that providing the relative power at several sensors reduces the MSE further. That makes us conclude that the relative gamma power holds the most information about short-term SWB and is distributed differently across the hemispheres, so that adding this distribution gives further information. The performed binomial test showed the superiority of the relative gamma power compared to the relative power of any other frequency band. That strengthens the importance of the relative gamma power distributed over the whole brain when it comes to determining short-term SWB. Considering the two left-handed participants, we see that for one participant, the relative power of the gamma band at all sensors yielded the lowest MSE, while for the other, it was the relative delta power. Again, we cannot make any generalized statements about left-handers as the number of left-handed participants is too low.

Input 3 used the relative power of all frequency bands at all channels and, thus, provided even more information to the k-NN algorithm. This approach lowered the MSE for seven participants. Most often, Input 2, using the relative gamma power at all sensors, yielded the lowest MSE. Since the relative gamma power outperformed the other relative powers in the last section, we conclude that adding additional relative power values adds more noise to the data and does not add any valuable information. This again is consistent with the relative gamma power holding the most information about short-term SWB.

In summary, we can say that the results differ on the individual participant level, but using the relative gamma power at all sensors as input for the k-NN seems to work reasonably well for most participants. Its importance was confirmed by a bivariate test that confirmed that the relative gamma power was way more often successful in predicting SWB than any other relative power. Also, when choosing just one sensor, the focus should be on higher frequency bands, as those tend to result in lower MSE for most participants. Including the relative power of all frequency bands at all channels does not improve the outcome; thus, we conclude that the relative power of the gamma band represents short-term SWB best.

#### 4.1.2. Linear Regression

When it comes to relative power and SWB or thermal comfort, many papers focus on a linear correlation. That is why we also performed a linear regression analysis to compare our results with findings in the literature to know if such a connection also holds for short-term SWB that might change every 30 s. After performing a linear regression for the relative power of different frequency bands at different sensors, we could not find any statistically significant results corrected for multiple comparisons; however, we want to report the results that showed significance for an uncorrected *p*-value < 0.05. We found an uncorrected significant negative correlation for the relative delta power when measured in the left frontal areas and the relative alpha power when measured in the temporal areas. What is surprising here is that the relative alpha power does not show any statistical correlation when measured on any frontal sensor. We expected that since a lot of research in the field of SWB or comfort focuses on the alpha frequency band in formal areas, and we also concluded in previous works that the alpha asymmetry of frontal areas is statistically significantly correlated to short-term SWB [9]. That seems to be a contradiction; however, we want to emphasize that two different measures were analyzed. FAA showed a statistically significant correlation to SWB, but the relative power, as calculated at a single sensor placed over the frontal lobe, did not. Hence, when focusing on the alpha frequency band in frontal areas, it is beneficial to use two sensors on opposite hemispheres and not just use the relative power at a single frontal sensor. Besides the location of the sensor, we find the alpha band to play an important role as it also gives the result with the lowest uncorrected *p*-value and thus can be seen as the most important. When moving to higher frequency bands, we see a positive linear correlation, which is in line with the literature and our own previous work [10], where we found that focusing on higher frequency bands usually gives the opposite direction of the linear regression when correlating its measure to short-term SWB. When focusing on the relative power of higher frequency bands, we find that the relative gamma power at four sensors is positively linearly correlated to short-term SWB. That is in accordance with the last section, where we found that the relative gamma power of several sensors provides information on how the SWB varies.

Summing up the sections that focus on relative power, we can answer RQ1 (see Introduction) as yes, the relative power of different frequency bands is correlated with short-term SWB. While the ideal parameter differs between participants, we can still conclude that such a correlation exists and that the relative gamma power holds the most information about the individually reported short-term SWB.

### 4.2. Time Series

Up to now, we extracted information from the EEG time series; now we use the time series themselves as input for the k-NN. We performed analyses for time series filtered into different frequency bands and also included an analysis without filtering into any frequency band to determine if filtering gives any benefit. Starting with Input 1, a single time series filtered in a specific frequency band at a certain channel, we already see that the unfiltered time series at a single channel gives the lowest MSE for many participants. That means that filtering into a frequency band might eliminate useful information for the k-NN algorithm. However, similar to the relative power approach in the last section, we see a high diversity between participants. Several combinations of frequency bands and sensors yield the best results for different participants. Comparing those results to Input 1 of the relative power analyses, we find that using the relative power of a frequency band at a specific channel outperforms the filtered time series in about 1/3 of the cases, which makes us conclude that while filtering the time series itself does not improve the results of the k-NN, calculating the relative power does.

Moving on to Input 2, where the filtered time series at all channels was used as the input, we see that filtering in the alpha band often yields the best results, followed by not filtering. The fact that not filtering the time series appears good again points to the ineffectiveness of filtering for that approach. Filtering in the gamma band and using all the time series as inputs seems to perform much worse than calculating the relative gamma power at each sensor.

Comparing all three inputs, we find that using a single time series at just one sensor gives the lowest MSE for all except one participant. That means that adding further time series seems to add noise and does not improve the performance of the k-NN. This is slightly different from the relative power approach, where adding the relative gamma power of all sensors improves performance. The reason for that might be that the time series itself is already highly dimensional, so adding a further time series increases the dimension drastically, whereas that is not the case if adding single relative power values.

### 4.3. Clustering

The main idea behind clustering the participants was to overcome the high diversity between participants. As we have already seen in our previous works [9,10], results can be found on group levels, but they vary hugely between individual participants. When looking at linear regression between either FAA or relative power and SWB, we see that the slope is statistically significant, e.g., positive on group level. However, we find that the slope for some individuals actually shows the opposite direction. As also discussed in the introduction section, several studies successfully group cohorts according to their EEG measures. We aimed to find clusters of people who have a similar relationship between their EEG measures and short-term SWB. We tried utilizing the individual distance between two participants by using a model trained for one participant and tested on the others. If the relationship between the two participants’ EEG measures and their short-term SWB is similar, we assume that the model trained for Participant A should work well on Participant B. We looked at partitions consisting of two, three, four, five, or six clusters. A priori, we were not sure which number would result in the best results. Using a total number of up to six clusters seemed reasonable because using more than six clusters when clustering 29 participants (excluding Participant 12 due to a lack of variability in the SWB values) would probably result in several clusters with just a single participant. If we look at the clusters that result when using the distance matrix from the linear regression of SWB to FAA as a model input, we find reasonable to good clustering as the Silhouette coefficient is between 0.4 and 0.7. A higher number of clusters results in a higher Silhouette coefficient, with k = 6 being the highest. When looking closer at that partition, we see that there is one cluster with just two participants and another one with just three participants. That might mean that six clusters might be too large to cluster our 29 participants. The partitions when using the distance metric from the relative power at a specific sensor and frequency with the k-NN approach as input give way lower Silhouette coefficients, which indicates that the diversity of the relative powers across participants is very high. However, when using a linear regression between the relative alpha power at T7 as input for the distance metric, we find a higher Silhouette coefficient again. This indicates that focusing on a specific frequency band and sensor, we can group the participants a lot better together than when allowing the input of any relative power at any sensor. Using the time series as an input shows the highest Silhouette coefficient for k = 4. This seems to be the most reasonable partition as each cluster contains at least four participants. It is also worth mentioning that excluding the two left-handed participants from the analyses did not really improve the Silhouette coefficient and that they very often do not belong to the same cluster. That can mean that we just have two left-handed participants that differ from each other, or also that there is no big difference between right-handed and left-handed participants in this analysis. However, since we only have two left-handed participants, we cannot make any definite conclusion.

Using the relative power often outperformed using the filtered time series as input for the k-NN for individual participants, but we found no clear clusters when using this relative power at a specific sensor as input for a distance metric. That might suggest that the relative power is most closely related to individual short-term SWB and clearly captures changes for individual participants, but this individuality then makes it difficult to generalize to more participants, so that no clusters can be found where participants within one cluster are closely related.

Now, let us move to the critical question of whether there are consistent clusters between methodologies, which would indicate that there are clusters of participants whose EEG measures correlate in a similar manner to short-term SWB. To answer RQ2 (see Section 1), we compared the clusters determined by the distance matrices calculated from models from either FAA, relative power at a sensor, or frequency band using k-NN, linear regression between the alpha power on T7 and SWB, or the time series filtered into a specific frequency band at a specific sensor and short-term SWB. As using the relative power with k-NN did not result in any proper partitions, it is of little surprise that its clustering does not align with the clustering of any other method. The clustering from FAA aligns with the T7 clustering for k = 4 clusters and with the time series algorithm for k = 2 clusters. That points to a similarity between participants when it comes to those EEG measures and their correlation with SWB. However, when comparing T7 to the time series method, we found adjusted Rand indices around 0, indicating no or hardly any overlap of the partitions when adjusted for random clustering. Thus, we answer RQ2 with no, as we could not find any underlying clusters of participants with a similar correlation between their EEG measures and the reported short-term SWB. This is the conclusion we can draw from our analyses. The reason why the clustering failed can be due to several reasons. The first is that the diversity of the participants is continuous and not discrete, as could be captured by clusters. The next reason might be that the measurements we extracted actually capture different underlying mechanisms. As they are all extracted from EEG data, we assumed the EEG to be the common ground, but as we extract different measures, we focus on different parts of the EEG data, and that might be the cause of not finding consistent clusters. The last reason could be due to the limitations of the participants in our study. We only included 30 participants. That definitely limits the number of clusters that can be analyzed. We included up to six clusters, so there is a possibility of a cluster structure, but it cannot be determined for 30 participants in our dataset.

Finally, we want to point out the limitations of our study. We included 30 college students, of whom 28 reported being right-handed. This is not a diverse cohort. Moreover, we performed the experiment in a controlled office environment. That means we cannot be sure that our findings hold for the general population, left-handed individuals, or how our findings would extrapolate to extreme conditions such as the freezing cold.

## 5. Conclusions

In this paper, we expanded our previous work on finding a correlation between EEG measures and short-term changes in SWB/comfort when varied via environmental changes. We first analyzed the correlation between relative power at different frequency bands and sensor locations. Using a k-NN approach, we found that using the relative power of the gamma band at all sensor locations gave the best results for most participants. If one is interested in determining SWB for a group without individualizing the setup, the relative gamma power over the whole brain is the best candidate. Moving to the linear regression, we found similar results to the literature, i.e., an opposite linear correlation between lower and higher frequency bands and SWB. We performed several *t*-tests, but no test survived the FDR-correction; however, uncorrelated significance was found for the relative power for sensors in the frontal and temporal areas of the left hemisphere. That means that the relative power is already reacting to short-term changes in SWB, such as 30 s intervals. While all those results show significance on a group level (*p*-value < 0.05 uncorrected), the specific parameters for individual participants vary. These results suggest that there is a linear correlation, but if one is interested in using it to determine the short-term SWB of a single person, it is advisable to calibrate the system for that specific user. In the last part of this manuscript, we focused on finding subgroups of participants with a similar correlation between EEG measures and short-term SWB. Finding such subgroups would give insight into different preferences of individuals and would make a real-life application easier, as users would only need to be aligned to a subgroup instead of calibrating the whole system. We wanted to make sure that the clusters capture the underlying structure of individuals’ correlations between EEG and SWB. That is why we included different measures and methods, such as FAA, relative power, or whole time series; however, no consistent clustering was found. While our sample size was just 30 participants, we conclude that there are most likely no clusters of individuals that can be consistently determined via different EEG measures, which will make it difficult to transfer a single model to an individual.

## Figures and Tables

**Table 1:** MSE for k-NN (k = 1) with the Input 2 for each participant and the relative power of a specific frequency band at all 14 sensors. Participants with Sub IDs 10 and 22 are left-handed; the rest are right-handed.

Sub ID	Band	MSE	Sub ID	Band	MSE	Sub ID	Band	MSE
1	gamma	0.64	11	theta	0.30	21	beta	0.72
2	gamma	1.64	13	gamma	6.66	22	delta	2.01
3	theta	1.91	14	gamma	0.82	23	theta	1.15
4	gamma	0.88	15	alpha	5.33	24	beta	4.04
5	alpha	1.37	16	gamma	0.77	25	gamma	2.51
6	gamma	6.15	17	gamma	0.45	26	gamma	0.81
7	gamma	0.98	18	beta	1.77	27	gamma	2.94
8	beta	1.13	19	gamma	0.21	28	gamma	2.01
9	delta	1.01	20	gamma	2.18	29	alpha	0.77
10	gamma	0.34				30	beta	2.28

**Table 2:** Overview of which input for the k-NN leads to the lowest MSE for each participant. The first column gives the participant ID, followed by the input leading to the lowest MSE. For Inputs 1 and 2, the parentheses give the specific frequency band and sensor or just the frequency band, respectively. Participants with Sub IDs 10 and 22 are left-handed; the rest are right-handed.

Sub ID	Input Nr.	Sub ID	Input Nr.	Sub ID	Input Nr.
1	2 (gamma)	11	1 (theta/FC6), 3	21	2 (beta)
2	3	13	1 (beta/O2)	22	3
3	3	14	2 (gamma)	23	2 (theta)
4	2 (gamma)	15	1 (theta/P7)	24	1 (delta/F7)
5	3	16	3	25	2 (gamma)
6	3	17	2 (gamma)	26	2 (gamma)
7	2 (gamma)	18	2 (beta)	27	2 (gamma)
8	1 (beta/AF4)	19	2 (gamma)	28	3
9	1 (gamma/F7)	20	1 (beta/T7)	29	2 (alpha)
10	2 (gamma)			30	3

**Table 3:** Overview of the two-sided *t*-test for the slope of the linear regression of relative power and SWB. The first column gives the frequency band for the relative power, the second column the channel, the third and fourth columns the mean slope and the standard deviation, respectively. This is followed by the Cohen’s d, the *p*-value, and the FDR-corrected *p*-value. The last two columns give the lower and upper bounds of the 95% confidence interval. The degree of freedom is 26 for all tests.

Band	Channel	k-Mean	Std	Cohen’s d	*p*-Value	*p*-Corr	CI_Low	CI_High
delta	AF3	−3.816	6.625	−0.565	0.0069	0.2053	−6.487	−1.145
	F7	−2.838	5.506	−0.506	0.0142	0.2053	−5.057	−0.618
alpha	T7	−5.772	9.996	−0.567	0.0067	0.2053	−9.802	−1.743
	T8	−4.315	9.243	−0.458	0.0249	0.2181	−8.041	−0.588
beta	F7	4.192	9.302	0.442	0.0299	0.2323	0.442	7.942
	FC5	4.279	8.607	0.488	0.0176	0.2053	0.809	7.748
gamma	AF3	6.959	14.878	0.459	0.0247	0.2181	0.961	12.956
	F7	6.418	12.168	0.518	0.0123	0.2053	1.513	11.323
	FC5	4.981	9.947	0.491	0.0169	0.2053	0.971	8.990
	T7	4.079	9.786	0.409	0.0432	0.3026	0.134	8.023

**Table 4:** Lowest MSE for the k-NN (k = 1) algorithm when using the time series filtered into a specific frequency band at all 14 sensors as input. The first column gives the Participant ID, the second column gives the specific frequency band, and the third column gives the calculated MSE. Participants with Sub ID 10s and 22 are left-handed; the rest are right-handed.

Sub ID	Band	MSE	Sub ID	Band	MSE	Sub ID	Band	MSE
1	alpha	1.31	11	non	0.32	21	alpha	2.66
2	alpha	3.18	13	gamma	8.99	22	gamma	2.87
3	alpha	2.04	14	non/beta/ gamma	1.56	23	beta	2.05
4	delta	1.33				24	beta	3.40
5	gamma	1.20	15	gamma	12.87	25	non	2.67
6	theta	10.80	16	alpha	0.85	26	delta	2.31
7	non	2.26	17	non	0.59	27	non	9.11
8	non	1.28	18	theta	3.61	28	delta	6.96
9	alpha	0.93	19	gamma	3.69	29	beta	0.97
10	theta	0.59	20	alpha	2.33	30	delta	6.66

**Table 5:** Adjusted Rand Index for pairwise comparison of the partitions. FAA_RP stands for the comparison between FAA and relative power; FAA_TS between FAA and time series; and RP_TS between relative power k-NN and time series. FAA_T7 compares FAA with the relative alpha power at T7, while RP_T7 and TS_T7 represent the comparisons with the relative power k-NN and the time series, respectively. The first column gives the number of clusters in the partition.

Clusters	FAA_RP	FAA_TS	RP_TS	FAA_T7	RP_T7	TS_T7
2	−0.047	0.603	−0.017	0.221	−0.017	0.153
3	0.020	0.222	0.016	0.106	0.036	−0.007
4	0.070	0.070	0.039	0.420	0.062	0.049
5	0.020	0.083	−0.001	0.215	0.002	0.012
6	−0.022	0.135	−0.010	0.248	0.019	−0.028

## Data Availability

The data presented in this study are available on request from the corresponding author.

## Supplementary Materials

The following supporting information can be downloaded at: https://www.mdpi.com/article/10.3390/s26020446/s1. Table S1: MSE for k-NN (k = 1), the input of the relative power of the delta band (0.5–3 Hz) of each sensor individually. The first column of each subtable gives the Subject ID and the number of different SWB values given by the participant in parentheses, the second column gives the chosen sensor, and the third column gives the calculated MSE. Light-gray-marked values are the lowest MSE for each participant for the relative power of the delta band in different sensors. Blue values show the lowest MSE compared to all inputs (compared to Tables S2 to S5). The two orange-marked participants are left-handed; the rest are right-handed. Table S2: Table entries are analogous to Table S1 but for the relative power of the theta band (4–7 Hz). Table S3: Table entries are analogous to Table S1 but for the relative power of the alpha band (8–13 Hz). Table S4: Table entries are analogous to Table S1 but for the relative power of the beta band (14–30 Hz). Table S5: Table entries are analogous to Table S1 but for the relative power of the gamma band (31–55 Hz). Table S6: MSE for k-NN (k = 1), the input of the relative power of a specific frequency band at all 14 sensors. The first column gives the specific frequency band; the second, fourth, and sixth columns give the Subject ID; and the third, fifth, and seventh columns are the calculated MSE. Light-gray-marked values are the lowest MSE for each participant over all frequency bands. The two orange-marked participants are left-handed; the rest are right-handed. Table S7: MSE for k-NN (k = 1), the input of the relative power of all five frequency bands at all 14 sensors. The first column in each subtable gives the Subject ID, followed by the calculated MSE and the number of different SWB values given by the participant. The two orange-marked participants are left-handed; the rest are right-handed. Table S8: Overview of the two-sided *t*-test for the slope of the linear regression of relative power and SWB. The first column gives the frequency band for the relative power, the second column the channel, and the third and fourth columns are the mean slope and the standard deviation, respectively. This is followed by statistics, the *p*-value, and the degrees of freedom. The last two columns give the lower and upper bounds of the 95% confidence interval. Bold-marked entries have a *p*-value lower than 0.1. Table S9: MSE for k-NN (k = 1), the input of the time series filtered into the delta band (0.5–3 Hz) of each sensor individually. The first column of each subtable gives the Subject ID and the number of different SWB values given by the participants in parentheses, the second column gives the sensor, and the third row gives the calculated MSE. Light-gray-marked values are the lowest MSE for each participant for the time series filtered into the delta band when comparing different sensors. Blue values show the lowest MSE compared to all inputs (compared to Tables S10–S14). The two orange-marked participants are left-handed; the rest are right-handed. Table S10: Table entries are analogous to Table S9 but for the time series filtered into the theta band (4–7 Hz). Table S11: Table entries are analogous to Table S9 but for the time series filtered into the alpha band (8–13 Hz). Table S12: Table entries are analogous to Table S9 but for the time series filtered into the beta band (14–30 Hz). Table S13: Table entries are analogous to Table S9 but for the time series filtered into the gamma band (31–55 Hz). Table S14: Table entries are analogous to Table S9, but for time series not filtered into any frequency band. Table S15: MSE for k-NN (k = 1), the input of the time series filtered into a specific frequency band (or not filtered) at all 14 sensors. The first column gives the specific frequency band; the second, fourth, and sixth columns give the Subject ID; and the third, fifth, and seventh columns give the calculated MSE. Light-gray-marked values are the lowest MSE for each participant over all frequency bands. The two orange-marked participants are left-handed; the rest are right-handed. Table S16: MSE for k-NN (k = 1), using the time series filtered into all five frequency bands at all 14 sensors as input. The first column in each subtable gives the Subject ID, followed by the calculated MSE and the number of different SWB values given by the participant. The two orange-marked participants are left-handed; the rest are right-handed. Table S17: MSE for k-NN (k = 1), using as input the time series filtered into all five frequency bands and not-filtered at all 14 sensors. The first column in each subtable gives the Subject ID, followed by the calculated MSE and the number of different SWB values given by the participant. The two orange-marked participants are left-handed; the rest are right-handed. Table S18: Label for each participant when using the distance matrix from the FAA algorithm and after Isomap embedding. The first column gives the subject ID. k indicates the number of clusters. The entries indicate the cluster number to which each subject belongs. SC gives the Silhouette coefficient for that clustering. The orange-marked ones are the left-handed participants. Table S19: Label for each participant when using the distance matrix from the relative power algorithm and after Isomap embedding. The first column gives the subject ID. k indicates the number of clusters. The entries indicate the cluster number to which each participant belongs. SC gives the Silhouette coefficient for that clustering. The orange-marked ones are the left-handed participants. Table S20: Label for each participant when using the distance matrix from the relative power linear regression algorithm and after Isomap embedding. The first column gives the subject ID. k indicates the number of clusters. The entries indicate the cluster number to which each participant belongs. SC gives the Silhouette coefficient for that clustering. The orange marked left are the left-handed participants. Table S21: Label for each participant when using the distance matrix from the time series algorithm and after Isomap embedding. The first column gives the subject ID. k indicates the number of clusters. The entries indicate the cluster number to which each participant belongs. SC gives the Silhouette coefficient for that clustering. The orange-marked ones are the left-handed participants.
