# Supplementary material for: Analysis of Short-Term Subjective Well-Being/Comfort and Its Correlation to Different EEG Metrics
Source: Sensors (Basel). 2026 Jan 9;26(2):446. doi: 10.3390/s26020446 (PMC12846148; doi:10.3390/s26020446)
Supplement: Supplementary file 1 [file sensors-26-00446-s001.zip › sensors-4042615-supplementary.pdf]

1. Relative power and k-nearest neighbors:

a. Relative power and k-nearest neighbors: Input 1

i. Relative power of the delta band (0.5–3 Hz)

Table S1: MSE for k-NN (k=1), the input of the relative power of the delta band (0.5–3 Hz) of each sensor individually. The first column of each subtable gives the Subject ID and the number of different SWB values given by the participant in parentheses. The second column indicates the chosen sensor, and the third column lists the calculated MSE. Light-gray-marked values are the lowest MSE for each participant for the relative power of the delta band when comparing different sensors. Values with an additional asterisk * show the lowest MSE comparing all inputs (compared to Tables S2 to S5). Participants with Sub IDs 10 and 22 are left-handed; the rest are right-handed.

Sub ID	Sensor	MSE	Sub ID	Sensor	MSE	Sub ID	Sensor	MSE	Sub ID	Sensor	MSE
1 (#3)	AF3	1.90	2 (#5)	AF3	6.20	3 (#6)	AF3	2.31	4 (#5)	AF3	2.38
	F7	1.80		F7	4.09		F7	4.45		F7	2.97
	F3	1.85		F3	4.31		F3	4.29		F3	2.22
	FC5	1.82		FC5	4.36		FC5	2.58		FC5	2.30
	T7	1.56		T7	5.36		T7	2.46		T7	2.84
	P7	1.87		P7	7.87		P7	3.06		P7	2.12
	O1	1.95		O1	6.18		O1	2.69		O1	3.55
	O2	1.61		O2	4.33		O2	3.57		O2	2.72
	P8	1.58		P8	2.76		P8	3.45		P8	1.90
	T8	1.69		T8	3.89		T8	4.77		T8	2.81
	FC6	2.01		FC6	4.56		FC6	3.80		FC6	3.06
	F4	1.64		F4	5.73		F4	3.65		F4	2.35
	F8	2.17		F8	3.89		F8	3.05		F8	2.55
	AF4	2.01		AF4	3.78		AF4	3.92		AF4	3.19
5 (#5)	AF3	1.66	6 (#10)	AF3	11.28	7 (#6)	AF3	3.09	8 (#6)	AF3	2.63
	F7	1.99		F7	11.77		F7	4.00		F7	2.28
	F3	2.10		F3	11.08		F3	2.94		F3	2.02
	FC5	1.74		FC5	10.58		FC5	3.33		FC5	1.47
	T7	2.22		T7	8.42		T7	2.06		T7	2.13
	P7	1.65		P7	8.81		P7*	1.56*		P7	1.83
	O1	2.07		O1	11.56		O1	2.50		O1	2.95

	O2	2.13		O2	10.02		O2	3.39		O2	2.57
	P8	2.03		P8	10.40		P8	2.41		P8	1.70
	T8	2.41		T8	10.51		T8	1.62		T8	2.22
	FC6	2.01		FC6	11.96		FC6	2.03		FC6	1.75
	F4	1.96		F4	10.34		F4	2.97		F4	1.70
	F8	1.97		F8	11.02		F8	3.72		F8	2.13
	AF4	1.75		AF4	10.49		AF4	3.50		AF4	2.58
9	AF3	1.58	10	AF3	0.56	11	AF3	0.38	13	AF3	6.67
(#4)	F7	1.88	(#4)	F7	0.57	(#3)	F7	0.38	(#10)	F7	7.70
	F3	1.66		F3	0.47		F3	0.54		F3	7.72
	FC5	1.72		FC5	0.63		FC5	0.39		FC5	9.18
	T7	1.85		T7	0.51		T7	0.40		T7	12.95
	P7	1.81		P7	0.82		P7	0.44		P7	9.49
	O1	1.67		O1	0.54		O1	0.49		O1	6.79
	O2	1.61		O2	0.61		O2	0.51		O2	9.75
	P8	1.54		P8	0.56		P8	0.47		P8	9.66
	T8	1.24		T8	0.63		T8	0.40		T8	9.70
	FC6	1.22		FC6	0.60		FC6	0.38		FC6	8.89
	F4	1.90		F4	0.67		F4	0.48		F4	8.75
	F8	1.69		F8	0.66		F8	0.49		F8	10.74
	AF4	1.87		AF4	0.67		AF4	0.48		AF4	10.49
14	AF3	3.21	15	AF3	5.63	16	AF3	1.55	17	AF3	0.74
(#5)	F7	2.89	(#6)	F7	7.31	(#4)	F7	1.73	(#3)	F7	0.80
	F3	3.21		F3	7.95		F3	1.18		F3	0.66
	FC5	2.53		FC5	5.64		FC5	1.60		FC5	0.73
	T7	3.10		T7	7.27		T7	1.48		T7	0.59
	P7	2.27		P7	6.63		P7	1.77		P7*	0.54*
	O1	3.48		O1	7.71		O1	1.92		O1	0.78
	O2	3.32		O2	6.70		O2	2.18		O2	0.62
	P8	2.06		P8	7.86		P8	1.56		P8	0.85
	T8	2.89		T8	6.36		T8	2.02		T8	0.86
	FC6	3.23		FC6	7.65		FC6	1.79		FC6	0.78
	F4	3.50		F4	6.93		F4	2.21		F4	0.74
	F8	3.16		F8	6.49		F8	2.26		F8	0.76
	AF4	3.44		AF4	6.23		AF4	1.87		AF4	0.97
18	AF3	5.12	19	AF3	4.88	20	AF3	1.87	21	AF3	1.35
(#6)	F7	5.68	(#5)	F7	3.44	(#6)	F7	1.98	(#6)	F7	1.51
	F3	4.77		F3	4.23		F3	2.47		F3	1.54
	FC5	3.49		FC5	5.18		FC5	2.48		FC5	1.51
	T7	4.77		T7	3.74		T7	1.93		T7	1.35
	P7	4.58		P7	4.19		P7	2.34		P7	1.04
	O1	6.40		O1	5.35		O1	2.24		O1	1.77

	O2	5.95		O2	3.58		O2	2.35		O2	1.32
	P8	5.35		P8	4.32		P8	2.36		P8	1.27
	T8	5.82		T8	3.08		T8	2.22		T8	1.76
	FC6	5.45		FC6	3.60		FC6	2.10		FC6	1.73
	F4	5.65		F4	3.75		F4	2.28		F4	1.25
	F8	6.38		F8	5.52		F8	1.94		F8	1.59
	AF4	4.77		AF4	3.47		AF4	2.06		AF4	1.68
22	AF3	4.01	23	AF3	2.03	24	AF3	4.78	25	AF3	3.58
(#7)	F7	3.67	(#4)	F7	2.10	(#7)	F7*	3.88*	(#6)	F7*	2.72*
	F3	3.97		F3	2.12		F3	5.33		F3	4.96
	FC5	4.67		FC5	2.42		FC5	3.90		FC5	4.31
	T7	3.46		T7	2.12		T7	5.16		T7	4.15
	P7	4.54		P7	2.03		P7	5.51		P7	4.99
	O1	3.46		O1	2.10		O1	4.57		O1	3.18
	O2	3.14		O2	1.55		O2	5.77		O2	3.76
	P8	3.72		P8	2.00		P8	4.66		P8	4.25
	T8	3.76		T8	2.08		T8	5.14		T8	4.73
	FC6	4.37		FC6	2.26		FC6	4.35		FC6	5.15
	F4	3.41		F4	2.15		F4	4.86		F4	3.78
	F8	3.04		F8	1.65		F8	5.67		F8	4.37
	AF4	2.95		AF4	2.07		AF4	4.94		AF4	5.37
26	AF3	1.96	27	AF3	8.93	28	AF3	3.37	29	AF3	1.65
(#6)	F7	1.99	(#7)	F7	10.39	(#6)	F7	3.28	(#3)	F7	1.21
	F3	1.81		F3	8.17		F3	6.46		F3	2.06
	FC5	1.89		FC5	8.76		FC5	4.19		FC5	1.51
	T7	1.45		T7	7.74		T7	5.51		T7	1.97
	P7	1.87		P7	9.14		P7	5.47		P7	1.56
	O1	1.96		O1	8.54		O1	5.85		O1	1.70
	O2	2.31		O2	7.24		O2	3.49		O2	1.24
	P8	2.18		P8	5.49		P8	4.54		P8	2.03
	T8	1.92		T8	8.33		T8	6.98		T8	1.20
	FC6	2.01		FC6	4.72		FC6	4.73		FC6	1.54
	F4	1.84		F4	9.65		F4	4.68		F4	1.61
	F8	1.85		F8	6.19		F8	5.70		F8	1.31
	AF4	2.23		AF4	6.29		AF4	4.06		AF4*	1.06*
30	AF3	6.49									
(#8)	F7	4.12									
	F3	4.32									
	FC5	4.27									
	T7	3.73									
	P7	2.28									
	O1	4.79									

	O2	5.32									
	P8	6.31									
	T8	5.29									
	FC6	5.21									
	F4	5.51									
	F8	5.77									
	AF4	4.82									

ii. Relative power of the theta band (4–7 Hz)

Table S2: Table entries are analogous to Table S1 but for the relative power of the theta band (4–7 Hz).

Sub ID	Sensor	MSE	Sub ID	Sensor	MSE	Sub ID	Sensor	MSE	Sub ID	Sensor	MSE
1 (#3)	AF3	1.37	2 (#5)	AF3	4.80	3 (#6)	AF3	3.43	4 (#5)	AF3	2.35
	F7	1.94		F7	3.73		F7	4.12		F7	1.67
	F3	1.92		F3	4.67		F3	3.06		F3	2.36
	FC5	1.54		FC5	3.38		FC5	3.49		FC5	2.06
	T7	2.50		T7	2.80		T7	3.12		T7	2.54
	P7	1.42		P7	4.96		P7	2.98		P7	2.72
	O1	1.79		O1	6.09		O1	4.06		O1	2.68
	O2	1.77		O2	4.62		O2	3.82		O2	1.51
	P8	1.80		P8	5.22		P8	3.48		P8	1.96
	T8	2.44		T8	4.87		T8	3.23		T8	2.19
	FC6	2.30		FC6	4.51		FC6	3.37		FC6	1.46
	F4	1.83		F4	4.60		F4	3.91		F4	2.12
	F8	2.12		F8	2.84		F8	4.23		F8	2.67
	AF4	1.80		AF4	6.11		AF4	4.00		AF4	2.16
5 (#5)	AF3	3.01	6 (#10)	AF3	12.28	7 (#6)	AF3	3.08	8 (#6)	AF3	3.38
	F7	1.87		F7	8.94		F7	3.48		F7	2.42
	F3	1.63		F3	12.67		F3	2.42		F3	2.38
	FC5	1.99		FC5	10.83		FC5	4.38		FC5	2.53
	T7	1.62		T7	11.72		T7	2.39		T7	2.43
	P7	1.28		P7	10.57		P7	2.19		P7	2.03
	O1	2.26		O1	11.03		O1	2.91		O1	3.22
	O2	2.25		O2	9.67		O2	3.59		O2	2.80
	P8	2.41		P8	8.58		P8	3.34		P8	2.22
	T8	2.12		T8	10.71		T8	2.95		T8	2.30
	FC6	2.51		FC6	10.03		FC6	3.16		FC6	2.20

	F4	1.97		F4	11.69		F4	3.66		F4	2.28
	F8	1.63		F8	12.59		F8	3.39		F8	3.07
	AF4	2.00		AF4	9.21		AF4	3.17		AF4	2.23
9	AF3	1.31	10	AF3	0.75	11	AF3	0.35	13	AF3	7.66
(#4)	F7	1.61	(#4)	F7	0.53	(#3)	F7	0.31	(#10)	F7	8.69
	F3	1.57		F3	0.60		F3	0.40		F3	8.98
	FC5	1.58		FC5	0.71		FC5	0.30		FC5	8.51
	T7	1.75		T7	0.59		T7	0.37		T7	9.89
	P7	1.52		P7	0.54		P7	0.39		P7	9.30
	O1	1.45		O1	0.66		O1	0.35		O1	7.03
	O2	1.78		O2	0.60		O2	0.35		O2	7.10
	P8	1.96		P8	0.65		P8	0.39		P8	8.23
	T8	1.22		T8	0.58		T8	0.37		T8	8.07
	FC6	2.43		FC6	0.74		FC6*	0.26*		FC6	11.9
	F4	1.49		F4	0.64		F4	0.37		F4	9.10
	F8	1.87		F8	0.82		F8	0.36		F8	7.56
	AF4	1.51		AF4	0.89		AF4	0.35		AF4	9.93
14	AF3	3.00	15	AF3	8.64	16	AF3	1.84	17	AF3	0.68
(#5)	F7	2.90	(#6)	F7	6.61	(#4)	F7	2.31	(#3)	F7	0.91
	F3	2.63		F3	6.89		F3	2.50		F3	0.89
	FC5	3.03		FC5	6.89		FC5	1.94		FC5	0.88
	T7	2.95		T7	5.51		T7	2.06		T7	0.86
	P7	2.26		P7*	4.64*		P7	1.94		P7	0.82
	O1	2.32		O1	8.58		O1	1.37		O1	1.34
	O2	2.65		O2	7.86		O2	2.02		O2	0.84
	P8	2.35		P8	8.71		P8	1.85		P8	0.85
	T8	2.19		T8	5.77		T8	1.45		T8	0.91
	FC6	3.82		FC6	7.02		FC6	2.11		FC6	0.81
	F4	3.05		F4	8.75		F4	1.53		F4	0.73
	F8	3.56		F8	9.04		F8	1.47		F8	0.93
	AF4	2.53		AF4	5.75		AF4	1.81		AF4	1.16
18	AF3	5.98	19	AF3	3.13	20	AF3	2.10	21	AF3	1.41
(#6)	F7	6.65	(#5)	F7	5.27	(#6)	F7	2.25	(#6)	F7	1.56
	F3	6.09		F3	4.68		F3	2.36		F3	1.44
	FC5	6.03		FC5	4.32		FC5	2.85		FC5	1.59
	T7	4.66		T7	4.35		T7	2.00		T7	1.85
	P7	4.46		P7	3.25		P7	2.01		P7	1.62
	O1	6.34		O1	4.29		O1	2.51		O1	1.26
	O2	5.69		O2	4.35		O2	2.05		O2	1.47
	P8	5.74		P8	2.91		P8	2.17		P8	1.46
	T8	4.14		T8	4.66		T8	2.38		T8	1.46
	FC6	6.43		FC6	4.66		FC6	2.57		FC6	1.84

	F4	6.02		F4	4.70		F4	2.36		F4	1.68
	F8	2.95		F8	2.91		F8	2.43		F8	1.56
	AF4	5.40		AF4	4.68		AF4	2.55		AF4	1.61
22	AF3	4.09	23	AF3	1.47	24	AF3	5.18	25	AF3	5.00
(#7)	F7	3.95	(#4)	F7	1.81	(#7)	F7	4.47	(#6)	F7	4.61
	F3	4.20		F3	1.72		F3	5.03		F3	5.01
	FC5	3.01		FC5	1.94		FC5	4.61		FC5	4.66
	T7	3.38		T7	2.07		T7	4.80		T7	3.57
	P7	4.00		P7	2.48		P7	5.05		P7	4.24
	O1	3.84		O1	2.40		O1	5.38		O1	4.88
	O2	4.16		O2	1.74		O2	4.84		O2	3.97
	P8	5.09		P8	2.25		P8	5.14		P8	3.72
	T8	4.03		T8	2.27		T8	5.38		T8	5.51
	FC6	3.22		FC6	1.67		FC6	5.34		FC6	3.21
	F4	3.70		F4	1.92		F4	5.65		F4	3.69
	F8	3.30		F8	1.65		F8	5.40		F8	5.15
	AF4	3.53		AF4	1.73		AF4	4.95		AF4	4.36
26	AF3	2.22	27	AF3	4.56	28	AF3	5.15	29	AF3	1.76
(#6)	F7	2.25	(#7)	F7	6.17	(#6)	F7	2.94	(#3)	F7	1.54
	F3	2.49		F3	5.87		F3	6.30		F3	1.89
	FC5	2.29		FC5	7.24		FC5	2.88		FC5	1.27
	T7	2.02		T7	7.89		T7	3.53		T7	1.89
	P7	2.37		P7	4.82		P7	3.58		P7	1.44
	O1	2.16		O1	6.16		O1	4.84		O1	1.62
	O2	2.03		O2	6.00		O2	3.20		O2	1.32
	P8	1.96		P8	8.14		P8	3.31		P8	1.51
	T8	2.10		T8	4.14		T8	5.32		T8	1.61
	FC6	2.56		FC6	7.59		FC6	4.10		FC6	1.68
	F4	2.43		F4	6.79		F4	3.91		F4	1.59
	F8	1.77		F8	8.55		F8	6.58		F8	1.63
	AF4	2.43		AF4	7.18		AF4	5.58		AF4	1.72
30	AF3	4.33									
(#8)	F7	5.37									
	F3	5.40									
	FC5	4.49									
	T7	5.01									
	P7	5.33									
	O1	3.97									
	O2	4.68									
	P8	4.21									
	T8	5.36									
	FC6	5.99									

	F4	6.18									
	F8	4.91									
	AF4	5.37									

iii. Relative power of the alpha band (8–13 Hz)

Table S3: Table entries are analogous to Table S1 but for the relative power of the alpha band (8–13 Hz).

Sub ID	Sensor	MSE	Sub ID	Sensor	MSE	Sub ID	Sensor	MSE	Sub ID	Sensor	MSE
1 (#3)	AF3	1.89	2 (#5)	AF3	4.47	3 (#6)	AF3	2.86	4 (#5)	AF3	3.33
	F7	1.79		F7	5.07		F7	2.91		F7	2.83
	F3	1.68		F3	3.18		F3	2.92		F3	2.46
	FC5	1.43		FC5	3.69		FC5	3.12		FC5	2.12
	T7	1.98		T7	4.93		T7	4.68		T7	2.09
	P7	1.86		P7	4.84		P7	3.40		P7	2.20
	O1	2.00		O1	4.64		O1	2.25		O1	2.62
	O2	2.07		O2	4.42		O2	2.86		O2	2.65
	P8	1.77		P8	4.62		P8*	2.20*		P8	3.25
	T8	1.55		T8	4.38		T8	3.11		T8	2.96
	FC6	1.87		FC6	4.62		FC6	2.97		FC6	2.61
	F4	1.99		F4	3.44		F4	3.40		F4	2.77
	F8	2.18		F8	2.73		F8	3.46		F8	2.28
	AF4	1.90		AF4	4.76		AF4	3.32		AF4	2.17
5 (#5)	AF3	2.18	6 (#10)	AF3	12.15	7 (#6)	AF3	3.44	8 (#6)	AF3	2.42
	F7	2.16		F7	11.32		F7	3.02		F7	2.50
	F3	2.47		F3	9.96		F3	2.77		F3	1.67
	FC5	2.22		FC5	12.39		FC5	3.16		FC5	1.77
	T7	1.65		T7	9.36		T7	3.16		T7	2.10
	P7	2.07		P7	8.18		P7	2.17		P7	2.20
	O1	2.49		O1	8.71		O1	3.20		O1	2.68
	O2	2.49		O2	13.59		O2	1.97		O2	2.25
	P8	1.84		P8	7.82		P8	3.44		P8	2.20
	T8	2.49		T8	8.83		T8	2.16		T8	1.72
	FC6	1.25		FC6	10.28		FC6	2.33		FC6	2.07
	F4	1.81		F4	10.82		F4	3.14		F4	2.68
	F8	1.75		F8	10.21		F8	3.27		F8	1.92
	AF4	2.62		AF4	10.12		AF4	2.67		AF4	2.18
9	AF3	1.33		AF3	0.58		AF3	0.57	13	AF3	8.36

(#4)	F7	1.40	10	F7	0.65	11	F7	0.52	(#10)	F7	7.46
	F3	1.57	(#4)	F3*	0.42*	(#3)	F3	0.36		F3	10.21
	FC5	1.85		FC5	0.49		FC5	0.27		FC5	10.44
	T7	1.97		T7	0.47		T7	0.37		T7	7.15
	P7	1.52		P7	0.75		P7	0.37		P7	8.08
	O1	1.45		O1	0.55		O1	0.35		O1	8.28
	O2	1.70		O2	0.71		O2	0.42		O2	7.98
	P8	1.79		P8	0.63		P8	0.35		P8	6.31
	T8	1.40		T8	0.65		T8	0.33		T8	8.03
	FC6	1.42		FC6	0.92		FC6	0.39		FC6	8.69
	F4	1.28		F4	0.56		F4	0.47		F4	9.75
	F8	1.40		F8	0.67		F8	0.44		F8	8.10
	AF4	2.12		AF4	0.67		AF4	0.39		AF4	8.26
14	AF3	3.31	15	AF3	6.68	16	AF3	1.82	17	AF3	0.57
(#5)	F7	3.27	(#6)	F7	8.79	(#4)	F7	2.06	(#3)	F7	0.80
	F3*	1.73*		F3	7.17		F3	1.79		F3	0.65
	FC5	2.66		FC5	8.17		FC5	2.27		FC5	0.85
	T7	3.65		T7	7.61		T7	2.48		T7	0.93
	P7	2.68		P7	5.80		P7	1.56		P7	0.74
	O1	2.97		O1	6.50		O1	2.05		O1	0.97
	O2	2.84		O2	7.07		O2	1.58		O2	0.72
	P8	3.84		P8	7.46		P8	1.69		P8	0.70
	T8	2.16		T8	8.85		T8	1.94		T8	0.73
	FC6	2.61		FC6	7.77		FC6	2.44		FC6	0.80
	F4	3.15		F4	7.24		F4	2.47		F4	0.77
	F8	3.11		F8	7.76		F8	1.85		F8	0.80
	AF4	2.82		AF4	7.56		AF4	1.87		AF4	0.80
18	AF3	2.98	19	AF3	4.42	20	AF3	2.11	21	AF3	1.42
(#6)	F7	5.45	(#5)	F7	4.47	(#6)	F7	2.21	(#6)	F7	1.22
	F3	7.00		F3	3.18		F3	2.32		F3	1.53
	FC5	6.85		FC5	3.75		FC5	2.20		FC5	1.56
	T7	2.97		T7	4.78		T7	2.26		T7	0.96
	P7	5.31		P7	3.92		P7	2.03		P7	1.19
	O1	6.26		O1	4.74		O1	2.39		O1	1.86
	O2	4.80		O2	3.73		O2	2.18		O2	1.47
	P8	4.03		P8	3.94		P8	2.26		P8	1.58
	T8	5.66		T8	2.79		T8	1.97		T8	1.40
	FC6	6.11		FC6	4.06		FC6	2.37		FC6	1.20
	F4	4.77		F4	4.06		F4	2.42		F4	1.24
	F8	5.58		F8	4.83		F8	2.29		F8	1.44
	AF4	5.83		AF4	3.45		AF4	2.19		AF4	1.35
	AF3	3.22		AF3	2.52		AF3	4.78		AF3	4.64

22	F7	3.83	23	F7	2.51	24	F7	4.69	25	F7	4.79
(#7)	F3	3.04	(#4)	F3	1.49	(#7)	F3	4.75	(#6)	F3	3.25
	FC5	4.66		FC5	2.28		FC5	5.23		FC5	5.04
	T7	3.97		T7	2.45		T7	5.90		T7	4.67
	P7	3.91		P7	2.13		P7	5.01		P7	5.22
	O1	4.47		O1	2.20		O1	4.90		O1	5.04
	O2	2.80		O2	2.39		O2	4.45		O2	5.51
	P8	3.67		P8	1.82		P8	5.31		P8	5.15
	T8	3.63		T8	2.47		T8	4.99		T8	4.12
	FC6	3.16		FC6	1.64		FC6	4.30		FC6	3.82
	F4	3.28		F4	1.74		F4	5.42		F4	3.12
	F8	4.82		F8	1.43		F8	5.52		F8	4.45
	AF4	2.97		AF4	2.19		AF4	5.86		AF4	3.99
26	AF3	2.16	27	AF3	4.49	28	AF3	3.77	29	AF3	1.45
(#6)	F7	2.42	(#7)	F7	6.40	(#6)	F7	5.95	(#3)	F7	2.07
	F3	2.57		F3	5.31		F3	6.22		F3	1.56
	FC5	2.30		FC5	4.83		FC5	5.98		FC5	1.59
	T7	1.92		T7	4.74		T7	6.62		T7	1.93
	P7	2.53		P7	8.23		P7	6.80		P7	1.35
	O1	2.02		O1	5.56		O1	6.12		O1	1.45
	O2	1.54		O2*	3.36*		O2	5.68		O2	1.25
	P8	1.76		P8	7.88		P8	4.22		P8	1.34
	T8	1.92		T8	5.51		T8	5.99		T8	1.90
	FC6	1.66		FC6	6.71		FC6	4.73		FC6	1.42
	F4	1.56		F4	5.34		F4	6.46		F4	1.52
	F8	1.88		F8	4.98		F8	6.19		F8	1.61
	AF4	1.68		AF4	5.17		AF4	6.36		AF4	1.86
30	AF3	5.09									
(#8)	F7	5.35									
	F3	4.53									
	FC5	4.62									
	T7	3.15									
	P7	5.9									
	O1	5.62									
	O2	4.13									
	P8	3.59									
	T8	5.00									
	FC6	4.78									
	F4	4.28									
	F8	5.40									
	AF4	3.59									

iv. Relative power of the beta band (14–30 Hz)

Table S4: Table entries are analogous to Table S1 but for the relative power of the beta band (14–30 Hz).

Sub ID	Sensor	MSE	Sub ID	Sensor	MSE	Sub ID	Sensor	MSE	Sub ID	Sensor	MSE
1	AF3	1.75	2	AF3	5.89	3	AF3	2.66	4	AF3	3.07
(#3)	F7	1.90	(#5)	F7	3.82	(#6)	F7	3.68	(#5)	F7	2.86
	F3	2.14		F3	5.09		F3	2.65		F3	2.14
	FC5	1.61		FC5	5.73		FC5	2.60		FC5	2.57
	T7	1.58		T7	5.04		T7	2.92		T7	2.62
	P7	1.38		P7	4.16		P7	2.89		P7	2.42
	O1	1.39		O1	4.40		O1	3.66		O1	2.23
	O2	1.58		O2	3.62		O2	2.92		O2	1.86
	P8	1.33		P8	3.69		P8	3.37		P8	2.16
	T8	2.05		T8	3.84		T8	2.62		T8	2.52
	FC6	1.57		FC6	3.47		FC6	2.85		FC6	2.88
	F4	1.94		F4	2.64		F4	2.98		F4	2.26
	F8	1.67		F8	4.24		F8	2.75		F8	2.80
	AF4	2.30		AF4	4.53		AF4	3.55		AF4	2.57
5	AF3	2.43	6	AF3	10.38	7	AF3	3.00	8	AF3	1.63
(#5)	F7	1.81	(#10)	F7	11.47	(#6)	F7	3.30	(#6)	F7	2.43
	F3	1.96		F3	11.62		F3	2.39		F3	2.33
	FC5	2.29		FC5	9.61		FC5	2.80		FC5	2.25
	T7	2.49		T7	11.19		T7	2.44		T7	1.75
	P7	2.10		P7	8.21		P7	2.92		P7	2.52
	O1	1.72		O1	12.33		O1	2.55		O1	2.05
	O2	1.93		O2	11.28		O2	3.61		O2	2.78
	P8	2.72		P8	7.98		P8	2.70		P8	1.98
	T8	2.50		T8	9.28		T8	2.03		T8	2.70
	FC6	2.00		FC6	11.61		FC6	3.20		FC6	1.43
	F4	2.29		F4	10.68		F4	3.48		F4	2.30
	F8	1.74		F8	9.78		F8	3.11		F8	2.53
	AF4	2.16		AF4	8.19		AF4	3.64		AF4*	1.05*
9	AF3	1.72	10	AF3	0.73	11	AF3	0.40	13	AF3	11.02
(#4)	F7	1.25	(#4)	F7	0.58	(#3)	F7	0.31	(#10)	F7	9.51
	F3	1.51		F3	0.71		F3	0.31		F3	11.15
	FC5	2.04		FC5	0.58		FC5	0.36		FC5	8.02
	T7	1.78		T7	0.64		T7	0.36		T7	10.77
	P7	1.46		P7	0.65		P7	0.43		P7	7.20
	O1	1.48		O1	0.63		O1	0.43		O1	8.20

	O2	1.90		O2	0.54		O2	0.30		O2*	5.49*
	P8	1.48		P8	0.51		P8	0.38		P8	10.18
	T8	1.66		T8	0.56		T8	0.39		T8	8.33
	FC6	1.79		FC6	0.56		FC6	0.33		FC6	9.66
	F4	1.72		F4	0.49		F4	0.36		F4	8.30
	F8	1.73		F8	0.53		F8	0.42		F8	7.25
	AF4	1.18		AF4	0.79		AF4	0.46		AF4	8.41
14	AF3	3.05	15	AF3	7.48	16	AF3	1.19	17	AF3	0.69
(#5)	F7	3.06	(#6)	F7	7.10	(#4)	F7	1.94	(#3)	F7	0.80
	F3	3.29		F3	5.38		F3	1.71		F3	0.91
	FC5	2.66		FC5	6.99		FC5	1.82		FC5	0.66
	T7	3.45		T7	7.69		T7	1.48		T7	0.68
	P7	3.84		P7	6.30		P7	1.74		P7	0.77
	O1	3.26		O1	8.01		O1	1.48		O1	0.66
	O2	2.94		O2	5.26		O2	1.37		O2	1.08
	P8	2.66		P8	6.99		P8	1.35		P8	0.78
	T8	3.81		T8	8.25		T8	2.31		T8	0.92
	FC6	3.23		FC6	8.01		FC6	2.03		FC6	0.93
	F4	1.90		F4	7.13		F4	1.95		F4	0.70
	F8	3.34		F8	5.25		F8	1.98		F8	1.07
	AF4	3.37		AF4	6.49		AF4	1.68		AF4	0.80
18	AF3	4.34	19	AF3	3.44	20	AF3	2.21	21	AF3	1.52
(#6)	F7	5.29	(#5)	F7	4.42	(#6)	F7	2.04	(#6)	F7	1.11
	F3	4.42		F3	3.55		F3	2.53		F3	1.28
	FC5	5.00		FC5	2.48		FC5	2.02		FC5	1.41
	T7	5.09		T7	3.32		T7*	1.84*		T7	1.15
	P7	4.40		P7	4.55		P7	2.25		P7	1.28
	O1	4.62		O1	3.75		O1	2.58		O1	1.57
	O2	5.02		O2	5.40		O2	2.36		O2	1.47
	P8	4.37		P8	2.94		P8	1.86		P8	1.72
	T8	4.15		T8	4.31		T8	2.14		T8*	0.93*
	FC6	5.38		FC6	4.26		FC6	2.20		FC6	1.33
	F4	3.71		F4	5.17		F4	2.43		F4	1.45
	F8	4.43		F8	4.62		F8	2.36		F8	1.32
	AF4*	2.51*		AF4	4.12		AF4	2.36		AF4	1.24
22	AF3	4.14	23	AF3	2.01	24	AF3	6.00	25	AF3	4.58
(#7)	F7	3.38	(#4)	F7	2.39	(#7)	F7	4.46	(#6)	F7	3.61
	F3	3.71		F3	2.10		F3	5.73		F3	3.72
	FC5	4.64		FC5	2.21		FC5	5.68		FC5	5.10
	T7	2.87		T7	2.22		T7	5.35		T7	6.00
	P7	4.01		P7	2.10		P7	5.32		P7	3.91
	O1	3.45		O1	2.31		O1	4.57		O1	3.52

	O2	3.37		O2	1.69		O2	4.48		O2	6.03
	P8	3.70		P8	1.74		P8	6.11		P8	3.64
	T8	2.96		T8	2.28		T8	6.11		T8	3.63
	FC6*	2.05*		FC6	1.83		FC6	4.33		FC6	3.16
	F4	3.38		F4*	1.34*		F4	4.23		F4	4.93
	F8	2.82		F8	1.48		F8	5.11		F8	3.76
	AF4	4.92		AF4	1.84		AF4	4.39		AF4	3.51
26 (#6)	AF3	2.16	27 (#7)	AF3	7.67	28 (#6)	AF3	3.00	29 (#3)	AF3	1.35
	F7	1.76		F7	7.63		F7	3.52		F7	1.37
	F3	2.10		F3	7.95		F3	4.07		F3	1.82
	FC5	1.40		FC5	5.47		FC5	3.26		FC5	1.32
	T7	1.35		T7	5.32		T7	3.41		T7	1.48
	P7	1.78		P7	9.17		P7	5.35		P7	1.51
	O1	1.65		O1	4.68		O1	5.80		O1	1.32
	O2	1.61		O2	6.69		O2	4.21		O2	1.08
	P8	1.51		P8	7.21		P8	6.35		P8	1.52
	T8	1.80		T8	8.11		T8	6.22		T8	1.35
	FC6	1.78		FC6	7.57		FC6	3.43		FC6	1.28
	F4	1.62		F4	7.22		F4	4.23		F4	1.65
	F8	1.76		F8	6.54		F8	4.48		F8	1.48
	AF4	1.91		AF4	5.61		AF4	4.94		AF4	1.31
30 (#8)	AF3	4.79									
	F7	4.18									
	F3	5.76									
	FC5	2.69									
	T7	2.42									
	P7	4.67									
	O1	6.65									
	O2	4.46									
	P8	5.15									
	T8	3.71									
	FC6	4.58									
	F4	6.23									
	F8	3.72									
	AF4	5.31									

v. Relative power of the gamma band (31–55 Hz)

Table S5: Table entries are analogous to Table S1 but for the relative power of the gamma band (31–55 Hz).

Sub ID	Sensor	MSE	Sub ID	Sensor	MSE	Sub ID	Sensor	MSE	Sub ID	Sensor	MSE
1	AF3	1.52	2	AF3	4.29	3	AF3	2.51	4	AF3	1.86
(#3)	F7	1.85	(#5)	F7	4.87	(#6)	F7	3.63	(#5)	F7	1.61
	F3	1.61		F3	4.42		F3	2.49		F3	1.51
	FC5	1.37		FC5	5.42		FC5	2.78		FC5	2.16
	T7	1.51		T7	5.13		T7	3.11		T7	2.14
	P7	1.27		P7	4.71		P7	2.97		P7	1.38
	O1	1.76		O1	6.02		O1	2.72		O1	1.72
	O2	1.71		O2	3.91		O2	2.65		O2*	1.19*
	P8	2.18		P8	2.56		P8	2.66		P8	1.30
	T8	1.54		T8*	1.91*		T8	3.65		T8	1.58
	FC6	1.35		FC6	2.00		FC6	4.32		FC6	2.17
	F4*	1.19*		F4	2.71		F4	3.97		F4	1.97
	F8	1.49		F8	2.64		F8	2.97		F8	2.04
	AF4	1.74		AF4	3.62		AF4	3.26		AF4	1.86
5	AF3	2.21	6	AF3	7.82	7	AF3	3.48	8	AF3	2.02
(#5)	F7	2.26	(#10)	F7*	7.42*	(#6)	F7	4.53	(#6)	F7	2.20
	F3	2.34		F3	9.22		F3	2.36		F3	1.67
	FC5	1.93		FC5	11.57		FC5	4.53		FC5	2.17
	T7*	1.81*		T7	8.25		T7	1.95		T7	2.37
	P7	2.28		P7	7.55		P7	2.30		P7	2.02
	O1	2.07		O1	10.08		O1	4.75		O1	1.50
	O2	2.28		O2	8.95		O2	3.62		O2	2.60
	P8	2.29		P8	7.71		P8	3.66		P8	2.17
	T8	2.01		T8	9.79		T8	2.98		T8	2.35
	FC6	2.41		FC6	11.93		FC6	2.66		FC6	2.00
	F4	2.16		F4	8.94		F4	3.19		F4	1.40
	F8	1.85		F8	10.08		F8	2.44		F8	2.50
	AF4	2.56		AF4	7.48		AF4	3.11		AF4	2.00
9	AF3	1.55	10	AF3	0.48	11	AF3	0.25	13	AF3	7.30
(#4)	F7*	0.97*	(#4)	F7	0.57	(#3)	F7	0.31	(#10)	F7	11.26
	F3	1.73		F3	0.67		F3	0.33		F3	10.82
	FC5	1.67		FC5	0.49		FC5	0.35		FC5	7.31
	T7	1.70		T7	0.53		T7	0.34		T7	7.79
	P7	1.66		P7	0.63		P7	0.31		P7	8.67
	O1	1.52		O1	0.57		O1	0.30		O1	9.93
	O2	1.85		O2	0.61		O2	0.35		O2	6.30
	P8	1.91		P8	0.81		P8	0.42		P8	6.56
	T8	1.37		T8	0.78		T8	0.38		T8	9.48
	FC6	1.67		FC6	0.58		FC6	0.29		FC6	10.8
	F4	1.87		F4	0.61		F4	0.40		F4	8.74

	F8	1.90		F8	0.46		F8	0.42		F8	8.43
	AF4	1.61		AF4	0.68		AF4	0.33		AF4	7.59
14	AF3	2.23	15	AF3	6.18	16	AF3	1.74	17	AF3	0.59
(#5)	F7	3.00	(#6)	F7	6.57	(#4)	F7	1.55	(#3)	F7	0.82
	F3	2.73		F3	8.48		F3	1.61		F3	0.62
	FC5	2.60		FC5	6.14		FC5	2.55		FC5	0.73
	T7	2.89		T7	7.86		T7	1.61		T7	0.57
	P7	4.11		P7	8.55		P7*	0.95*		P7	0.84
	O1	3.97		O1	5.81		O1	1.10		O1	0.82
	O2	3.52		O2	6.93		O2	1.34		O2	0.64
	P8	2.05		P8	6.58		P8	1.24		P8	0.80
	T8	2.34		T8	6.00		T8	1.94		T8	0.74
	FC6	3.27		FC6	7.44		FC6	1.40		FC6	0.81
	F4	4.08		F4	7.18		F4	1.74		F4	0.78
	F8	3.29		F8	7.67		F8	2.02		F8	0.81
	AF4	2.50		AF4	7.51		AF4	1.11		AF4	0.85
18	AF3	5.00	19	AF3*	1.09*	20	AF3	2.17	21	AF3	1.47
(#6)	F7	6.29	(#5)	F7	3.12	(#6)	F7	2.21	(#6)	F7	1.37
	F3	4.91		F3	2.51		F3	2.09		F3	1.44
	FC5	5.51		FC5	3.31		FC5	2.14		FC5	1.27
	T7	6.51		T7	3.39		T7	2.54		T7	1.55
	P7	4.97		P7	3.21		P7	2.46		P7	1.75
	O1	5.65		O1	3.94		O1	2.21		O1	1.12
	O2	4.26		O2	3.52		O2	2.35		O2	1.30
	P8	4.65		P8	3.95		P8	2.45		P8	1.37
	T8	5.40		T8	4.22		T8	2.14		T8	1.55
	FC6	6.34		FC6	4.70		FC6	2.32		FC6	1.48
	F4	3.57		F4	3.40		F4	2.32		F4	1.63
	F8	4.57		F8	4.21		F8	2.08		F8	1.25
	AF4	6.20		AF4	3.12		AF4	2.24		AF4	1.30
22	AF3	3.87	23	AF3	2.24	24	AF3	5.62	25	AF3	4.99
(#7)	F7	4.92	(#4)	F7	1.71	(#7)	F7	4.96	(#6)	F7	4.48
	F3	3.54		F3	2.26		F3	4.97		F3	4.45
	FC5	3.86		FC5	1.75		FC5	5.70		FC5	2.99
	T7	3.29		T7	1.82		T7	5.10		T7	3.75
	P7	4.18		P7	1.82		P7	6.29		P7	3.46
	O1	3.16		O1	1.79		O1	4.51		O1	4.58
	O2	3.78		O2	1.99		O2	4.91		O2	5.25
	P8	3.82		P8	1.89		P8	5.57		P8	5.63
	T8	3.07		T8	1.58		T8	5.59		T8	5.12
	FC6	2.97		FC6	1.72		FC6	5.02		FC6	4.31
	F4	4.29		F4	2.09		F4	5.94		F4	4.63

	F8	3.08		F8	2.07		F8	4.72		F8	4.96
	AF4	4.21		AF4	2.37		AF4	4.98		AF4	5.42
26	AF3	1.67	27	AF3	6.24	28	AF3*	2.78*	29	AF3	1.21
(#6)	F7	1.30	(#7)	F7	9.11	(#6)	F7	3.53	(#3)	F7	1.54
	F3	1.20		F3	8.11		F3	5.43		F3	1.56
	FC5*	1.03*		FC5	6.36		FC5	4.04		FC5	1.70
	T7	1.26		T7	6.83		T7	2.89		T7	1.83
	P7	1.22		P7	7.36		P7	6.14		P7	1.48
	O1	1.13		O1	6.17		O1	5.35		O1	1.89
	O2	1.63		O2	4.69		O2	3.23		O2	1.30
	P8	1.78		P8	7.36		P8	4.27		P8	1.35
	T8	1.28		T8	6.56		T8	3.95		T8	1.62
	FC6	1.41		FC6	6.60		FC6	4.52		FC6	1.94
	F4	1.11		F4	6.62		F4	3.85		F4	1.62
	F8	1.09		F8	5.69		F8	4.86		F8	1.32
	AF4	1.38		AF4	6.10		AF4	5.36		AF4	1.73
30	AF3	4.83									
(#8)	F7	4.36									
	F3	5.96									
	FC5	4.42									
	T7*	2.17*									
	P7	6.10									
	O1	5.27									
	O2	4.14									
	P8	5.50									
	T8	6.03									
	FC6	5.62									
	F4	6.47									
	F8	5.69									
	AF4	5.50									

b. Relative power and k-nearest neighbors: Input 2

Table S6: MSE for k-NN (k=1), the input of the relative power of a specific frequency band at all 14 sensors. The first column gives the specific frequency band; the second, fourth, and sixth columns give the Subject ID; and the third, fifth, and seventh columns list the calculated MSE. Light-gray-marked values are the lowest MSE for each participant across all frequency bands. The two orange-marked participants are left-handed; the rest are right-handed.

Band	Sub ID	MSE	Sub ID	MSE	Sub ID	MSE
0–3	1 (#3)	1.17	11 (#3)	0.40	21 (#6)	1.09
4–7		1.24		0.30		1.30
8–13		1.23		0.33		1.28
14–30		0.99		0.45		0.72
31–55		0.64		0.33		0.87
0–3	2 (#5)	2.80	12 (#1)	0	22 (#7)	2.01
4–7		2.96		0		4.01
8–13		4.29		0		3.61
14–30		2.09		0		2.46
31–55		1.64		0		2.30
0–3	3 (#6)	2.46	13 (#10)	8.48	23 (#4)	1.51
4–7		1.91		9.38		1.15
8–13		2.12		9.75		2.10
14–30		3.62		8.64		1.85
31–55		2.54		6.66		1.35
0–3	4 (#5)	2.51	14 (#5)	0.92	24 (#7)	4.42
4–7		2.07		1.95		4.38
8–13		1.84		2.73		4.71
14–30		2.59		1.56		4.04
31–55		0.88		0.82		4.05
0–3	5 (#5)	1.43	15 (#6)	5.64	25 (#6)	3.27
4–7		1.65		6.88		5.42
8–13		1.37		5.33		3.79
14–30		1.79		7.76		3.31
31–55		1.40		8.25		2.51
0–3	6 (#10)	9.63	16 (#4)	1.26	26 (#6)	1.70
4–7		8.89		1.60		1.84
8-13		7.56		1.13		1.44
14–30		7.94		0.82		1.37
31–55		6.15		0.77		0.81
0–3	7 (#6)	1.50	17 (#3)	0.54	27 (#7)	5.15
4–7		2.39		0.74		5.98
8–13		1.47		0.92		4.02
14–30		2.06		0.88		6.01
31–55		0.98		0.45		2.94
0–3	8 (#6)	1.80	18 (#6)	5.11	28 (#6)	2.46
4–7		1.53		4.83		2.86
8–13		1.75		5.06		5.05
14–30		1.13		1.77		2.99
31–55		1.53		2.71		2.10

0–3	9 (#4)	1.01	19 (#5)	2.19	29 (#3)	1.51
4–7		1.54		3.60		1.14
8–13		1.40		3.44		0.77
14–30		1.30		1.66		1.13
31–55		1.07		0.21		1.08
0–3	10 (#4)	0.36	20 (#6)	2.32	30 (#8)	3.06
4–7		0.74		2.42		3.24
8–13		0.44		2.59		3.44
14–30		0.38		2.46		1.81
31–55		0.34		2.18		2.28

c. Relative power and k-nearest neighbors: Input 3

Table S7: MSE for k-NN (k=1), the input of the relative power of all five frequency bands at all 14 sensors. The first column in each subtable gives the Subject ID, followed by the calculated MSE and the number of different SWB values given by the participant. Participants with Sub ID 10 and 22 are left-handed; the rest are right-handed.

Sub ID	MSE	#SWB	Sub ID	MSE	#SWB	Sub ID	MSE	#SWB	Sub ID	MSE	#SWB
1	0.75	3	9	1.28	4	18	4.25	6	26	1.22	6
2	1.42	4	10	0.44	4	19	1.01	5	27	6.30	7
3	1.49	6	11	0.26	3	20	2.44	6	28	1.70	6
4	2.00	5	13	7.34	10	21	0.98	6	29	0.80	3
5	1.32	5	14	1.13	5	22	1.91	7	30	1.97	8
6	5.62	10	15	5.21	6	23	1.26	4			
7	1.28	6	16	0.63	4	24	4.72	7			
8	1.20	6	17	0.62	3	25	3.06	6			

2. Relative power and SWB: Linear regression

Table S8: Overview of the two-sided t-test for the slope of the linear regression of relative power and SWB. The first column gives the frequency band for the relative power, the second column lists the channel, and the third and fourth columns are the mean slope and the standard deviation, respectively. This is followed by the statistic, Cohen's d, the p-value, the FDR-corrected p-value, and the degrees of freedom. The last two columns give the lower and upper bounds of the 95% confidence interval. Bold-marked entries have a p-value lower than 0.1.

Band	Channel	K_mean	Std	Cohen's d	Stat	P_value	P_corr	Df	Ci_low	Ci_high
0–3	**AF3**	**-3.816**	**6.625**	**-0.565**	**-2.937**	**0.0069**	**0.2053**	**26**	**-6.487**	**-1.145**
	**F7**	**-2.838**	**5.506**	**-0.506**	**-2.628**	**0.0142**	**0.2053**	**26**	**-5.057**	**-0.618**
	F3	-1.269	5.703	-0.218	-1.135	0.2668	0.6520	26	-3.568	1.030
	**FC5**	**-1.968**	**5.028**	**-0.384**	**-1.996**	**0.0565**	**0.3597**	**26**	**-3.995**	**0.059**
	T7	-1.158	5.930	-0.192	-0.995	0.3287	0.7321	26	-3.548	1.233
	P7	-1.616	9.552	-0.166	-0.863	0.3961	0.7495	26	-5.467	2.234
	O1	1.131	8.814	0.126	0.654	0.5187	0.7981	26	-2.422	4.684
	O2	0.252	10.561	0.023	0.122	0.9040	0.9445	26	-4.005	4.510
	P8	-0.897	9.503	-0.093	-0.482	0.6342	0.8376	26	-4.728	2.934
	T8	0.481	6.533	0.072	0.375	0.7105	0.8896	26	-2.153	3.114
	FC6	-0.929	6.35	-0.144	-0.746	0.4624	0.7895	26	-3.489	1.631
	F4	-1.247	7.231	-0.169	-0.879	0.3872	0.7495	26	-4.162	1.668
	F8	-0.197	5.312	-0.036	-0.189	0.8515	0.9445	26	-2.338	1.944
	AF4	-1.244	6.147	-0.199	-1.032	0.3117	0.7273	26	-3.722	1.234
4–7	AF3	-0.599	10.134	-0.058	-0.301	0.7657	0.9084	26	-4.684	3.487
	F7	-3.166	12.601	-0.247	-1.281	0.2115	0.6169	26	-8.246	1.914
	F3	-0.684	9.812	-0.068	-0.355	0.7253	0.8896	26	-4.639	3.272
	FC5	-2.864	11.868	-0.237	-1.230	0.2295	0.6427	26	-7.648	1.920
	T7	-2.353	12.694	-0.182	-0.945	0.3534	0.7493	26	-7.470	2.765
	P7	-1.887	16.155	-0.115	-0.596	0.5565	0.7981	26	-8.400	4.625
	O1	4.661	15.310	0.299	1.553	0.1326	0.5461	26	-1.510	10.833
	O2	2.045	13.029	0.154	0.800	0.4308	0.7550	26	-3.208	7.297
	P8	-0.358	11.675	-0.030	-0.156	0.8771	0.9445	26	-5.064	4.349
	T8	-3.119	13.752	-0.223	-1.156	0.2581	0.6520	26	-8.663	2.425
	FC6	0.425	10.826	0.038	0.200	0.8430	0.9445	26	-3.940	4.789
	F4	0.861	11.106	0.076	0.395	0.6958	0.8896	26	-3.616	5.338
	F8	-0.486	11.319	-0.042	-0.219	0.8283	0.9445	26	-5.049	4.077
	AF4	0.188	9.559	0.019	0.100	0.9210	0.9480	26	-3.666	4.041
8–13	AF3	-0.205	8.013	-0.025	-0.131	0.8970	0.9445	26	-3.436	3.025
	F7	-0.968	7.942	-0.120	-0.621	0.5398	0.7981	26	-4.169	2.234
	F3	-1.155	7.371	-0.154	-0.799	0.4314	0.7550	26	-4.127	1.816
	FC5	-2.827	9.492	-0.292	-1.519	0.1409	0.5481	26	-6.653	0.999
	**T7**	**-5.772**	**9.996**	**-0.567**	**-2.945**	**0.0067**	**0.2053**	**26**	**-9.802**	**-1.743**
	P7	-3.630	11.551	-0.308	-1.602	0.1212	0.5461	26	-8.286	1.027
	**O1**	**-2.848**	**7.880**	**-0.355**	**-1.843**	**0.0767**	**0.4150**	**26**	**-6.025**	**0.328**
	O2	-0.283	9.019	-0.031	-0.160	0.8741	0.9445	26	-3.919	3.353
	P8	-1.927	8.675	-0.218	-1.132	0.2678	0.6520	26	-5.424	1.570
	**T8**	**-4.315**	**9.243**	**-0.458**	**-2.380**	**0.0249**	**0.2181**	**26**	**-8.041**	**-0.588**
	FC6	-1.744	9.049	-0.189	-0.983	0.3347	0.7321	26	-5.392	1.903
	F4	-0.988	8.215	-0.118	-0.613	0.5450	0.7981	26	-4.300	2.324
	**F8**	**-3.259**	**9.218**	**-0.347**	**-1.803**	**0.0830**	**0.4150**	**26**	**-6.976**	**0.457**
	AF4	-1.703	7.705	-0.217	-1.127	0.2701	0.6520	26	-4.809	1.403
14–30	**AF3**	**3.973**	**11.185**	**0.349**	**1.811**	**0.0817**	**0.4150**	**26**	**-0.536**	**8.482**
	**F7**	**4.192**	**9.302**	**0.442**	**2.298**	**0.0299**	**0.2323**	**26**	**0.442**	**7.942**
	F3	2.483	8.869	0.275	1.427	0.1654	0.5513	26	-1.093	6.058
	**FC5**	**4.279**	**8.607**	**0.488**	**2.535**	**0.0176**	**0.2053**	**26**	**0.809**	**7.748**
	T7	2.609	10.038	0.255	1.325	0.1966	0.6081	26	-1.438	6.656

	P7	4.017	15.571	0.253	1.316	0.1998	0.6081	26	-2.260	10.294
	O1	-0.034	11.467	-0.003	-0.015	0.9881	0.9901	26	-4.657	4.589
	O2	0.028	11.274	0.002	0.013	0.9901	0.9901	26	-4.517	4.573
	P8	1.354	13.835	0.096	0.499	0.6220	0.8373	26	-4.223	6.931
	T8	1.861	10.27	0.178	0.924	0.3640	0.7493	26	-2.279	6.001
	FC6	1.201	11.078	0.106	0.553	0.5850	0.8190	26	-3.264	5.667
	F4	1.747	12.383	0.138	0.719	0.4783	0.7972	26	-3.245	6.739
	F8	1.205	10.374	0.114	0.592	0.5587	0.7981	26	-2.977	5.387
	AF4	1.720	9.962	0.169	0.880	0.3867	0.7495	26	-2.296	5.736
31–55	**AF3**	**6.959**	**14.878**	**0.459**	**2.385**	**0.0247**	**0.2181**	**26**	**0.961**	**12.956**
	**F7**	**6.418**	**12.168**	**0.518**	**2.690**	**0.0123**	**0.2053**	**26**	**1.513**	**11.323**
	F3	3.873	13.430	0.283	1.470	0.1534	0.5513	26	-1.541	9.280
	**FC5**	**4.981**	**9.947**	**0.491**	**2.553**	**0.0169**	**0.2053**	**26**	**0.971**	**8.990**
	**T7**	**4.079**	**9.786**	**0.409**	**2.125**	**0.0432**	**0.3026**	**26**	**0.134**	**8.023**
	P7	3.613	12.714	0.279	1.449	0.1593	0.5513	26	-1.513	8.738
	O1	0.590	10.857	0.053	0.277	0.7839	0.9146	26	-3.787	4.967
	O2	-0.861	12.703	-0.067	-0.346	0.7325	0.8896	26	-5.982	4.260
	P8	1.739	13.779	0.124	0.643	0.5256	0.7981	26	-3.816	7.293
	T8	3.792	12.314	0.302	1.570	0.1285	0.5461	26	-1.172	8.756
	FC6	0.836	12.559	0.065	0.339	0.7371	0.8896	26	-4.227	5.899
	F4	2.28	13.793	0.162	0.843	0.4069	0.7496	26	-3.28	7.840
	F8	1.247	12.577	0.097	0.505	0.6175	0.8373	26	-3.824	6.317
	AF4	1.86	14.836	0.123	0.639	0.5282	0.7981	26	-4.121	7.841

3. Time series and k-nearest neighbors:

a. Time series and k-nearest neighbors: Input 1

i. Time series filtered into the delta band (0.5–3 Hz)

Table S9: MSE for k-NN (k=1), the input of the time series filtered into the delta band (0.5–3 Hz) of each sensor individually. The first column of each subtable gives the Subject ID and the number of different SWB values given by the participants in parentheses, the second column lists the sensor, and the third column indicates the calculated MSE. Light-gray-marked values are the lowest MSE for each participant for the time series filtered into the delta band when comparing different sensors. Values marked with an asterisk * are the lowest MSE comparing all inputs (compared to Tables S10 to S14). Participants with Sub IDs 10 and 22 are left-handed; the rest are right-handed.

Sub ID	Sensor	MSE	Sub ID	Sensor	MSE	Sub ID	Sensor	MSE	Sub ID	Sensor	MSE
1 (#3)	AF3	1.77	2 (#5)	AF3	6.72	3 (#6)	AF3	2.55	4 (#5)	AF3	1.99
	F7	1.62		F7	5.84		F7	1.95		F7	2.17
	F3	1.86		F3	6.20		F3	2.31		F3	1.39
	FC5	2.03		FC5	5.20		FC5	2.21		FC5	1.88
	T7	1.55		T7	3.22		T7	2.07		T7	1.81
	P7	1.61		P7	4.64		P7	2.34		P7	2.53
	O1	1.85		O1	4.66		O1	2.44		O1	2.08
	O2	1.34		O2	5.40		O2	2.20		O2	2.54
	P8	1.79		P8	5.16		P8	3.25		P8	2.08
	T8	1.76		T8	4.86		T8	1.68		T8	1.50
	FC6	1.44		FC6	5.64		FC6	2.08		FC6	1.92
	F4	1.80		F4	5.38		F4	2.62		F4	1.86
	F8	2.07		F8	4.80		F8	2.13		F8	1.97
	AF4	1.79		AF4	4.42		AF4	2.41		AF4*	1.38*
5 (#5)	AF3	1.84	6 (#10)	AF3	15.96	7 (#6)	AF3	3.09	8 (#6)	AF3	2.75
	F7	1.43		F7	15.35		F7	2.03		F7	3.00
	F3	2.38		F3	14.06		F3	4.54		F3	3.39
	FC5	1.70		FC5	14.25		FC5	1.94		FC5	2.25
	T7	1.57		T7	12.00		T7	2.30		T7	2.10
	P7	1.61		P7	13.23		P7	2.48		P7	2.69
	O1	2.32		O1	12.35		O1	3.54		O1	1.93
	O2	1.57		O2	14.25		O2	2.61		O2	2.48
	P8	2.93		P8	14.83		P8	2.62		P8	1.60
	T8	1.76		T8	12.91		T8	2.74		T8	2.12
	FC6	1.99		FC6	19.06		FC6	2.52		FC6	3.01
	F4	1.97		F4	12.17		F4	3.33		F4	3.12
	F8	1.39		F8	11.43		F8	4.59		F8	2.15
	AF4	1.84		AF4	16.56		AF4	2.52		AF4	2.40
9 (#4)	AF3	1.27	10 (#4)	AF3	0.98	11 (#3)	AF3	0.47	13 (#10)	AF3	13.57
	F7	1.11		F7	1.00		F7	0.53		F7	14.66
	F3	1.89		F3	0.98		F3	0.52		F3	10.21
	FC5	1.34		FC5	0.88		FC5	0.57		FC5	12.78
	T7	1.51		T7	1.14		T7	0.65		T7	9.56
	P7	1.17		P7	1.25		P7	0.59		P7	17.03
	O1	1.11		O1	1.17		O1	0.45		O1	17.46
	O2	1.20		O2	0.72		O2	0.49		O2	9.99
	P8	1.28		P8	0.75		P8	0.53		P8	12.72
	T8	1.31		T8	0.79		T8	0.55		T8	13.56
	FC6	1.46		FC6	0.75		FC6	0.51		FC6	12.76
	F4	1.20		F4	0.58		F4	0.56		F4	9.94

	F8	1.00		F8	0.63		F8	0.49		F8	12.74
	AF4	1.11		AF4	0.67		AF4	0.47		AF4	15.41
14	AF3	3.90	15	AF3	17.14	16	AF3	1.05	17	AF3	1.00
(#5)	F7	1.94	(#6)	F7	9.59	(#4)	F7	1.62	(#3)	F7	1.05
	F3	4.26		F3	9.87		F3	1.45		F3	0.88
	FC5	4.31		FC5	9.48		FC5	2.78		FC5	0.95
	T7	3.37		T7	11.97		T7	2.07		T7	1.23
	P7	2.76		P7	9.01		P7	1.27		P7	0.89
	O1	3.10		O1	10.67		O1	2.56		O1	0.84
	O2	3.81		O2	13.32		O2	2.24		O2	1.15
	P8	3.69		P8	13.55		P8	1.24		P8	1.00
	T8	2.16		T8	14.52		T8	2.45		T8	0.99
	FC6	3.71		FC6	9.74		FC6	2.09		FC6	0.81
	F4	2.04		F4	9.14		F4	1.67		F4	0.92
	F8	2.81		F8	15.68		F8	1.33		F8	0.76
	AF4	3.68		AF4	17.14		AF4	1.00		AF4	0.85
18	AF3	3.70	19	AF3	6.25	20	AF3	3.03	21	AF3	2.89
(#6)	F7	3.69	(#5)	F7	4.72	(#6)	F7	3.63	(#6)	F7	3.21
	F3	3.84		F3	8.97		F3	2.15		F3	2.61
	FC5	3.07		FC5	5.44		FC5	3.01		FC5	2.54
	T7	4.46		T7	7.42		T7	2.46		T7	2.33
	P7	4.78		P7	5.48		P7	3.99		P7	3.85
	O1	5.25		O1	6.21		O1	3.18		O1	3.30
	O2	5.34		O2	4.08		O2	4.21		O2	2.70
	P8	5.39		P8	7.07		P8	3.89		P8	2.19
	T8	3.33		T8	6.89		T8	2.71		T8	3.32
	FC6	3.16		FC6	6.49		FC6	3.09		FC6	3.14
	F4	3.99		F4	7.73		F4	3.19		F4	2.56
	F8	8.60		F8	6.17		F8	3.84		F8	2.96
	AF4	6.54		AF4	8.68		AF4	2.97		AF4	2.81
22	AF3	3.57	23	AF3	2.19	24	AF3	4.95	25	AF3	5.22
(#7)	F7	5.61	(#4)	F7	2.18	(#7)	F7	5.99	(#6)	F7	5.71
	F3	3.66		F3	1.85		F3	5.49		F3	4.93
	FC5	4.00		FC5	2.07		FC5	5.03		FC5	5.41
	T7	3.69		T7	2.15		T7	4.89		T7	4.34
	P7	5.04		P7	1.84		P7	5.46		P7	6.01
	O1	4.53		O1	2.14		O1	4.36		O1	3.47
	O2	4.45		O2	2.19		O2	4.78		O2	3.78
	P8	3.75		P8	1.93		P8	5.03		P8	2.81
	T8	3.68		T8	1.98		T8	5.64		T8	4.73
	FC6	3.25		FC6	2.44		FC6	5.20		FC6	4.85
	F4	2.71		F4	2.25		F4	6.05		F4	4.96

	F8	3.18		F8	2.35		F8	4.61		F8	4.95
	AF4	2.86		AF4	2.07		AF4	5.32		AF4	4.36
26	AF3	2.30	27	AF3	10.99	28	AF3	6.11	29	AF3	1.51
(#6)	F7	3.05	(#7)	F7	8.98	(#6)	F7	6.68	(#3)	F7	1.79
	F3	2.33		F3	9.45		F3	7.14		F3	1.59
	FC5	2.21		FC5	9.20		FC5	7.02		FC5	1.55
	T7	2.82		T7	8.48		T7	4.84		T7	1.92
	P7	2.59		P7	8.28		P7	5.91		P7	1.44
	O1	2.65		O1	8.71		O1	5.36		O1	1.49
	O2	2.28		O2	9.12		O2	5.85		O2	1.20
	P8	2.48		P8	11.66		P8	6.05		P8	1.41
	T8	2.70		T8	13.96		T8	7.44		T8	1.87
	FC6	2.46		FC6	9.44		FC6	4.89		FC6	1.36
	F4	2.20		F4	9.24		F4	6.4		F4	1.07
	F8	2.14		F8	11.32		F8	6.99		F8	1.79
	AF4	2.35		AF4	10.78		AF4	6.49		AF4	0.96
30	AF3	5.14									
(#8)	F7*	4.79*									
	F3	7.10									
	FC5	6.07									
	T7	10.44									
	P7	8.05									
	O1	12.74									
	O2	11.23									
	P8	7.04									
	T8	10.49									
	FC6	5.05									
	F4	9.10									
	F8	5.99									
	AF4	6.21									

ii. Time series filtered into the theta band (4–7 Hz)

Table S10: Table entries are analogous to Table S9 but for the time series filtered into the theta band (4–7 Hz).

Sub ID	Sensor	MSE	Sub ID	Sensor	MSE	Sub ID	Sensor	MSE	Sub ID	Sensor	MSE
1	AF3	1.62	2	AF3	5.46	3	AF3	3.24	4	AF3	3.11
(#3)	F7	2.01	(#5)	F7	4.82	(#6)	F7	3.27	(#5)	F7	3.33
	F3	1.54		F3	4.38		F3	3.20		F3	2.64
	FC5	1.75		FC5	4.96		FC5	2.68		FC5	3.15

	T7	1.76		T7	4.38		T7	2.24		T7	3.00
	P7	1.48		P7	5.54		P7	2.21		P7	2.29
	O1	1.55		O1	4.48		O1	2.76		O1	2.28
	O2	1.46		O2	5.46		O2	2.83		O2	3.21
	P8	1.46		P8	4.84		P8	2.49		P8	3.24
	T8	1.28		T8	4.18		T8	1.97		T8	2.18
	FC6	1.79		FC6	2.86		FC6	2.39		FC6	2.63
	F4	1.72		F4	4.10		F4	3.00		F4	2.17
	F8	2.06		F8	4.04		F8	2.45		F8	2.11
	AF4	1.45		AF4	4.10		AF4	1.99		AF4	1.82
5	AF3	2.03	6	AF3	13.26	7	AF3	3.81	8	AF3	2.25
(#5)	F7	1.31	(#10)	F7	15.23	(#6)	F7	1.96	(#6)	F7	2.25
	F3	2.11		F3	12.48		F3	5.54		F3	2.37
	FC5	1.93		FC5	11.62		FC5	2.77		FC5	2.25
	T7	1.54		T7	10.49		T7	3.41		T7	2.28
	P7	1.19		P7	14.78		P7	2.87		P7	3.72
	O1	1.62		O1	13.25		O1	5.45		O1	2.07
	O2	1.28		O2	13.46		O2	3.59		O2	1.70
	P8	2.93		P8	11.58		P8	3.59		P8*	1.24*
	T8	1.78		T8	10.68		T8	3.04		T8	1.61
	FC6	1.72		FC6	13.22		FC6	4.04		FC6	2.88
	F4	1.42		F4	12.31		F4	5.26		F4	3.01
	F8	1.38		F8	10.11		F8	5.96		F8	2.49
	AF4	1.85		AF4	15.74		AF4*	1.87*		AF4	2.81
9	AF3	1.24	10	AF3	0.64	11	AF3	0.53	13	AF3	12.38
(#4)	F7	1.42	(#4)	F7	0.93	(#3)	F7	0.39	(#10)	F7	13.93
	F3	1.83		F3	0.69		F3	0.55		F3	14.00
	FC5	1.97		FC5	1.00		FC5	0.41		FC5	17.15
	T7	1.10		T7	0.81		T7	0.44		T7	10.16
	P7	1.17		P7	0.93		P7	0.41		P7	10.09
	O1	1.44		O1	1.01		O1	0.49		O1	15.15
	O2	1.80		O2	0.74		O2	0.47		O2	14.25
	P8	1.51		P8	0.72		P8	0.53		P8	14.03
	T8	1.82		T8	0.79		T8	0.56		T8	17.88
	FC6	1.63		FC6	0.77		FC6	0.52		FC6	17.22
	F4	1.72		F4	0.84		F4	0.60		F4	14.24
	F8	1.82		F8	0.68		F8	0.61		F8	16.90
	AF4	1.21		AF4	0.79		AF4	0.55		AF4	13.41
14	AF3	3.49	15	AF3	19.49	16	AF3	1.04	17	AF3	1.40
(#5)	F7	2.16	(#6)	F7	8.86	(#4)	F7	1.18	(#3)	F7	0.91
	F3	4.53		F3	8.16		F3	1.16		F3	0.59
	FC5	4.60		FC5	7.07		FC5	2.51		FC5	0.99

	T7	3.44		T7	8.52		T7	1.95		T7	1.41
	P7	3.15		P7	8.52		P7	1.33		P7	1.03
	O1	2.13		O1	9.39		O1	2.29		O1	0.92
	O2	2.16		O2	12.59		O2	2.58		O2	1.23
	P8	3.47		P8	10.57		P8	1.31		P8	0.84
	T8	3.37		T8*	6.38*		T8	1.71		T8	1.19
	FC6	3.06		FC6	7.97		FC6	2.18		FC6	0.73
	F4	1.78		F4	10.23		F4	1.22		F4	1.07
	F8	2.72		F8	13.42		F8	1.27		F8	0.72
	AF4	3.88		AF4	19.55		AF4	1.00		AF4	0.92
18	AF3	4.60	19	AF3	6.01	20	AF3	3.20	21	AF3	3.14
(#6)	F7	4.06	(#5)	F7	5.38	(#6)	F7	3.15	(#6)	F7	4.86
	F3	3.36		F3	5.76		F3	2.26		F3	2.76
	FC5	4.28		FC5	5.18		FC5	3.42		FC5	3.13
	T7	5.52		T7	8.31		T7	2.45		T7	2.44
	P7	5.60		P7	5.49		P7	3.54		P7	4.40
	O1	6.15		O1	5.72		O1	3.57		O1	4.34
	O2	5.75		O2*	3.39*		O2	4.73		O2	2.70
	P8	5.49		P8	4.28		P8	3.73		P8	2.08
	T8	4.36		T8	6.34		T8	3.19		T8	3.75
	FC6	4.91		FC6	6.35		FC6	2.55		FC6	3.46
	F4	4.27		F4	6.31		F4	2.34		F4	2.74
	F8	4.90		F8	5.86		F8	3.90		F8	3.24
	AF4	6.49		AF4	5.66		AF4	3.32		AF4	3.31
22	AF3	3.97	23	AF3	2.18	24	AF3	5.38	25	AF3	4.89
(#7)	F7	3.52	(#4)	F7	2.49	(#7)	F7	5.71	(#6)	F7	5.47
	F3	3.70		F3	2.64		F3	4.66		F3	5.04
	FC5	3.83		FC5	2.31		FC5	5.00		FC5	5.95
	T7	4.19		T7	2.27		T7	3.55		T7	4.88
	P7	6.56		P7	2.20		P7	5.53		P7	6.36
	O1	3.75		O1	2.44		O1	5.21		O1	3.82
	O2	3.60		O2	2.40		O2	6.03		O2	3.38
	P8	4.12		P8	2.42		P8	4.42		P8	3.03
	T8	4.95		T8	2.39		T8	6.89		T8	4.1
	FC6	4.79		FC6	2.48		FC6	5.39		FC6	3.85
	F4	4.53		F4	2.32		F4	6.13		F4	3.68
	F8	2.79		F8	2.56		F8	4.83		F8	3.30
	AF4	3.64		AF4	2.61		AF4	5.80		AF4	4.22
26	AF3	2.35	27	AF3	12.21	28	AF3	5.84	29	AF3	1.40
(#6)	F7	2.75	(#7)	F7	8.28	(#6)	F7	6.46	(#3)	F7	2.11
	F3	3.28		F3	8.39		F3	6.63		F3	1.87
	FC5	3.15		FC5	8.25		FC5	6.59		FC5	1.59

	T7	2.48		T7	7.70		T7*	3.25*		T7	1.89
	P7	2.96		P7	8.51		P7	5.64		P7	2.17
	O1	2.88		O1	11.79		O1	7.21		O1	1.47
	O2	2.51		O2	11.09		O2	5.67		O2	1.67
	P8	3.09		P8	7.85		P8	7.06		P8	1.89
	T8	2.86		T8	8.06		T8	7.41		T8	2.32
	FC6	2.99		FC6	8.36		FC6	4.23		FC6	1.85
	F4	2.44		F4	11.49		F4	7.19		F4	1.21
	F8	2.96		F8	8.72		F8	6.99		F8	1.97
	AF4	2.62		AF4	7.98		AF4	6.28		AF4	0.87
30 (#8)	AF3	6.52									
	F7	6.14									
	F3	9.93									
	FC5	8.11									
	T7	12.95									
	P7	6.81									
	O1	12.45									
	O2	13.22									
	P8	8.41									
	T8	13.4									
	FC6	8.40									
	F4	8.53									
	F8	7.40									
	AF4	8.78									

iii. Time series filtered into the alpha band (8–13 Hz)

Table S11: Table entries are analogous to Table S9 but for the time series filtered into the alpha band (8–13 Hz).

Sub ID	Sensor	MSE	Sub ID	Sensor	MSE	Sub ID	Sensor	MSE	Sub ID	Sensor	MSE
1 (#3)	AF3	1.21	2 (#5)	AF3	3.84	3 (#6)	AF3	2.17	4 (#5)	AF3	2.88
	F7	1.73		F7	5.16		F7	2.11		F7	3.00
	F3	1.13		F3	3.02		F3	2.48		F3	3.08
	FC5	1.31		FC5	4.74		FC5	2.18		FC5	2.75
	T7	1.32		T7	4.92		T7	1.94		T7	3.13
	P7	1.31		P7	5.52		P7	2.08		P7	2.51
	O1	1.39		O1	5.22		O1	4.49		O1	2.28
	O2	1.24		O2	5.32		O2	3.54		O2	2.56

	P8	1.45		P8	4.70		P8	3.11		P8	2.28
	T8	1.58		T8	4.16		T8	2.08		T8	2.61
	FC6*	1.04*		FC6	3.38		FC6	1.80		FC6	2.94
	F4	1.23		F4	3.24		F4	3.30		F4	2.76
	F8	1.24		F8	4.38		F8	1.92		F8	2.04
	AF4	1.27		AF4	3.46		AF4	2.01		AF4	2.57
5	AF3	1.69	6	AF3	13.95	7	AF3	3.75	8	AF3	3.21
(#5)	F7	1.26	(#10)	F7	14.60	(#6)	F7	2.71	(#6)	F7	2.84
	F3	1.72		F3	13.72		F3	5.74		F3	3.54
	FC5	1.73		FC5	9.59		FC5	2.58		FC5	2.85
	T7	1.59		T7	9.72		T7	3.20		T7	2.55
	P7	1.16		P7	14.15		P7	2.74		P7	4.33
	O1	2.03		O1	9.98		O1	3.88		O1	2.52
	O2	1.34		O2	12.20		O2	2.83		O2	1.99
	P8	4.03		P8	12.59		P8	4.67		P8	1.52
	T8	1.78		T8	15.51		T8	4.07		T8	2.42
	FC6	1.42		FC6	20.59		FC6	3.90		FC6	3.43
	F4	1.14		F4	12.46		F4	4.88		F4	3.52
	F8	1.19		F8	12.57		F8	5.52		F8	2.37
	AF4	1.45		AF4	18.19		AF4	2.54		AF4	2.37
9	AF3*	0.79*	10	AF3	0.73	11	AF3	0.60	13	AF3	14.84
(#4)	F7	0.93	(#4)	F7	1.07	(#3)	F7	0.57	(#10)	F7	15.53
	F3	1.38		F3	0.96		F3	0.64		F3	13.35
	FC5	1.08		FC5	0.95		FC5	0.51		FC5	11.56
	T7	1.03		T7	0.95		T7	0.43		T7	10.63
	P7	0.94		P7	1.25		P7	0.49		P7	10.43
	O1	0.99		O1	1.09		O1	0.51		O1	12.93
	O2	1.72		O2	1.17		O2	0.59		O2	9.29
	P8	1.75		P8	1.15		P8	0.55		P8	9.99
	T8	1.66		T8	0.88		T8	0.57		T8	12.44
	FC6	0.94		FC6	0.77		FC6	0.60		FC6	14.43
	F4	1.34		F4	0.75		F4	0.69		F4	13.24
	F8	1.41		F8	0.75		F8	0.64		F8	16.51
	AF4	1.07		AF4	0.85		AF4	0.65		AF4	13.65
14	AF3	3.44	15	AF3	24.67	16	AF3	0.84	17	AF3	0.84
(#5)	F7	2.62	(#6)	F7	9.86	(#4)	F7	0.93	(#3)	F7	1.05
	F3	4.63		F3	9.62		F3	1.13		F3	0.65
	FC5	4.06		FC5	11.86		FC5	2.45		FC5	0.85
	T7	3.32		T7	8.58		T7	1.93		T7	1.55
	P7	3.15		P7	10.64		P7	1.31		P7	0.97
	O1*	1.53*		O1	9.04		O1	2.18		O1	0.85
	O2	1.85		O2	12.54		O2	1.78		O2	0.97

	P8	3.16		P8	12.54		P8	1.13		P8	1.13
	T8	3.97		T8	7.36		T8	1.69		T8	1.23
	FC6	4.04		FC6	9.59		FC6	2.13		FC6	0.81
	F4	1.65		F4	7.83		F4	1.60		F4	0.96
	F8	3.00		F8	12.84		F8*	0.82*		F8	0.91
	AF4	3.22		AF4	21.72		AF4	1.07		AF4	0.77
18	AF3	4.67	19	AF3	7.49	20	AF3	2.67	21	AF3	2.26
(#6)	F7	4.27	(#5)	F7	5.62	(#6)	F7	3.25	(#6)	F7	4.46
	F3*	2.82*		F3	6.69		F3	2.09		F3	2.04
	FC5	3.19		FC5	5.72		FC5	2.67		FC5	3.40
	T7	5.10		T7	8.37		T7	2.04		T7	2.49
	P7	4.88		P7	4.58		P7	4.73		P7	4.14
	O1	6.61		O1	6.03		O1	2.40		O1	3.79
	O2	7.30		O2	5.39		O2	2.99		O2	2.35
	P8	4.04		P8	5.51		P8	3.13		P8	2.09
	T8	3.66		T8	8.11		T8	3.41		T8	3.47
	FC6	3.37		FC6	7.21		FC6	2.78		FC6	2.31
	F4	4.10		F4	7.41		F4	3.07		F4	2.82
	F8	4.28		F8	7.77		F8	3.59		F8	2.85
	AF4	4.79		AF4	8.10		AF4	2.78		AF4	3.24
22	AF3	3.32	23	AF3	2.20	24	AF3	5.07	25	AF3	3.89
(#7)	F7	3.75	(#4)	F7	2.16	(#7)	F7	4.63	(#6)	F7	5.32
	F3	4.00		F3	2.30		F3	4.88		F3	3.93
	FC5	3.87		FC5	2.20		FC5	5.89		FC5	6.26
	T7	4.55		T7	1.92		T7	4.63		T7	3.27
	P7	5.32		P7	1.99		P7	5.93		P7	3.90
	O1	3.26		O1	2.53		O1	6.38		O1	4.48
	O2	3.75		O2	2.03		O2	4.70		O2	2.90
	P8	4.69		P8	2.20		P8	4.88		P8	2.55
	T8	4.56		T8	2.26		T8	3.43		T8	2.99
	FC6	3.40		FC6	2.15		FC6	4.72		FC6	3.88
	F4	3.97		F4	2.30		F4	5.01		F4	5.47
	F8	3.10		F8	2.31		F8	4.66		F8	4.30
	AF4	3.58		AF4	2.03		AF4	5.07		AF4	4.29
26	AF3	2.61	27	AF3	10.4	28	AF3	7.22	29	AF3	1.75
(#6)	F7	2.50	(#7)	F7	9.94	(#6)	F7	7.32	(#3)	F7	2.00
	F3	2.99		F3	8.99		F3	7.38		F3	1.69
	FC5	2.96		FC5	9.68		FC5	7.94		FC5	1.53
	T7	2.65		T7	8.40		T7	3.80		T7	1.99
	P7*	2.04*		P7	8.44		P7	5.58		P7	1.67
	O1	2.62		O1	9.15		O1	6.73		O1	1.44
	O2	2.38		O2	9.50		O2	6.22		O2	2.11

	P8	2.96		P8	7.81		P8	3.65		P8	1.80
	T8	2.31		T8	7.88		T8	7.51		T8	1.97
	FC6	2.91		FC6	8.57		FC6	3.99		FC6	1.63
	F4	2.72		F4	9.81		F4	7.10		F4	1.89
	F8	2.80		F8	9.35		F8	6.81		F8	1.36
	AF4	2.91		AF4	9.29		AF4	6.47		AF4	1.00
30 (#8)	AF3	6.66									
	F7	7.88									
	F3	7.44									
	FC5	8.19									
	T7	16.08									
	P7	6.48									
	O1	10.4									
	O2	8.33									
	P8	6.32									
	T8	12.89									
	FC6	8.74									
	F4	9.32									
	F8	8.93									
	AF4	5.88									

iv. Time series filtered into the beta band (14–30 Hz)

Table S12: Table entries are analogous to Table S9 but for the time series filtered into the beta band (14–30 Hz).

Sub ID	Sensor	MSE	Sub ID	Sensor	MSE	Sub ID	Sensor	MSE	Sub ID	Sensor	MSE
1 (#3)	AF3	2.00	2 (#5)	AF3	6.54	3 (#6)	AF3	2.97	4 (#54)	AF3	2.69
	F7	1.90		F7	5.96		F7	2.38		F7	2.67
	F3	2.06		F3	6.84		F3	3.83		F3	2.00
	FC5	1.85		FC5	4.30		FC5	2.72		FC5	2.56
	T7	1.56		T7	5.08		T7	2.17		T7	1.67
	P7	1.51		P7	5.50		P7	2.13		P7	1.93
	O1	1.96		O1	4.00		O1	3.08		O1	1.69
	O2	1.51		O2	4.56		O2	4.11		O2	2.36
	P8	1.73		P8	4.70		P8	2.61		P8	2.22
	T8	1.93		T8	4.84		T8	2.69		T8	1.76
	FC6	1.56		FC6	5.22		FC6	2.62		FC6	2.06
	F4	1.55		F4	6.30		F4	2.83		F4	1.86
	F8	2.01		F8	3.14		F8	2.83		F8	1.88

	AF4	1.45		AF4	3.26		AF4	2.55		AF4	1.79
5	AF3	1.77	6	AF3	15.10	7	AF3	3.51	8	AF3	5.48
(#5)	F7	1.38	(#10)	F7	11.31	(#6)	F7	2.23	(#6)	F7	2.49
	F3	2.54		F3	15.23		F3	4.90		F3	3.79
	FC5	1.47		FC5	9.85		FC5	2.64		FC5	1.46
	T7	1.59		T7	8.21		T7	2.61		T7	2.12
	P7*	1.07*		P7	14.72		P7	2.64		P7	5.19
	O1	1.31		O1	13.04		O1	3.39		O1	1.33
	O2	1.11		O2	12.33		O2	3.42		O2	1.55
	P8	3.03		P8	14.05		P8	3.65		P8	1.31
	T8	1.65		T8	13.88		T8	2.46		T8	1.34
	FC6	1.51		FC6	17.80		FC6	3.42		FC6	3.33
	F4	1.50		F4	11.84		F4	4.80		F4	1.79
	F8	1.54		F8	12.62		F8	4.91		F8	3.76
	AF4	1.35		AF4	17.28		AF4	2.61		AF4	1.66
9	AF3	1.15	10	AF3	1.01	11	AF3	0.45	13	AF3	11.21
(#4)	F7	1.14	(#4)	F7	1.03	(#3)	F7	0.39	(#10)	F7	10.65
	F3	2.37		F3	1.17		F3	0.49		F3	14.46
	FC5	1.24		FC5	1.27		FC5	0.48		FC5	10.78
	T7	0.96		T7	1.00		T7	0.37		T7	12.62
	P7	1.04		P7	1.23		P7	0.40		P7*	7.76*
	O1	1.13		O1	1.23		O1	0.51		O1	9.09
	O2	1.63		O2	0.99		O2	0.57		O2	9.40
	P8	1.24		P8	1.31		P8	0.55		P8	11.44
	T8	1.37		T8	1.30		T8	0.53		T8	12.13
	FC6	1.13		FC6	1.10		FC6	0.41		FC6	12.38
	F4	1.58		F4	0.75		F4	0.49		F4	15.54
	F8	1.13		F8	1.04		F8	0.47		F8	13.19
	AF4	0.99		AF4	0.91		AF4	0.48		AF4	11.10
14	AF3	4.24	15	AF3	26.48	16	AF3	1.16	17	AF3	0.73
(#5)	F7	1.60	(#6)	F7	6.62	(#4)	F7	1.24	(#3)	F7	0.93
	F3	5.43		F3	9.52		F3	1.75		F3	0.88
	FC5	5.04		FC5	8.38		FC5	2.60		FC5	0.76
	T7	3.25		T7	7.94		T7	1.71		T7	1.12
	P7	2.51		P7	8.12		P7	1.33		P7	0.87
	O1	1.54		O1	11.43		O1	1.18		O1	0.65
	O2	1.57		O2	10.81		O2	1.42		O2	0.63
	P8	1.72		P8	9.25		P8	1.31		P8	0.53
	T8	2.72		T8	6.70		T8	1.51		T8	0.75
	FC6	3.26		FC6	8.49		FC6	2.24		FC6	0.59
	F4	1.65		F4	6.62		F4	1.33		F4	0.87
	F8	3.22		F8	13.22		F8	1.29		F8	0.72

	AF4	4.15		AF4	25.51		AF4	0.95		AF4	0.77
18	AF3	3.27	19	AF3	6.00	20	AF3	4.48	21	AF3	6.55
(#6)	F7	3.37	(#5)	F7	6.86	(#6)	F7	4.05	(#6)	F7	7.90
	F3	3.34		F3	7.14		F3*	1.72*		F3	2.33
	FC5	3.37		FC5	5.92		FC5	4.52		FC5	5.07
	T7	5.84		T7	7.65		T7	1.96		T7	2.07
	P7	5.18		P7	4.66		P7	5.26		P7	4.53
	O1	4.82		O1	6.23		O1	3.33		O1	6.09
	O2	4.76		O2	4.56		O2	3.06		O2	3.37
	P8	4.73		P8	3.52		P8	3.22		P8*	1.66*
	T8	5.12		T8	7.07		T8	3.07		T8	2.92
	FC6	4.06		FC6	5.96		FC6	3.46		FC6	3.47
	F4	6.09		F4	7.07		F4	3.96		F4	2.75
	F8	6.82		F8	5.66		F8	3.35		F8	3.41
	AF4	6.84		AF4	5.55		AF4	2.49		AF4	3.77
22	AF3	7.03	23	AF3*	1.50*	24	AF3	4.18	25	AF3	3.01
(#7)	F7	7.01	(#4)	F7	1.75	(#7)	F7	4.38	(#6)	F7	6.29
	F3	5.71		F3	1.64		F3	3.38		F3	3.33
	FC5	9.84		FC5	1.81		FC5	4.33		FC5	6.70
	T7	4.60		T7	1.66		T7	4.67		T7	3.15
	P7	7.86		P7	1.98		P7	3.93		P7	3.12
	O1	3.22		O1	2.70		O1	7.80		O1	4.00
	O2	3.60		O2	2.43		O2	5.11		O2	2.82
	P8	5.48		P8	2.14		P8	4.03		P8	2.81
	T8	8.25		T8	2.11		T8	3.17		T8	2.42
	FC6	5.62		FC6	1.73		FC6	4.12		FC6	3.42
	F4	5.23		F4	1.93		F4	3.78		F4	2.96
	F8	7.34		F8	1.84		F8	3.68		F8	2.88
	AF4	5.23		AF4	1.80		AF4	3.93		AF4	2.78
26	AF3	2.24	27	AF3	10.6	28	AF3	6.73	29	AF3	1.20
(#6)	F7	2.76	(#7)	F7	9.20	(#6)	F7	7.30	(#3)	F7	1.25
	F3	3.42		F3	8.18		F3	6.52		F3	1.53
	FC5	3.10		FC5	9.11		FC5	7.95		FC5	1.59
	T7	2.61		T7	8.98		T7	3.48		T7	2.00
	P7	2.69		P7	9.15		P7	7.77		P7	1.43
	O1	3.69		O1	12.08		O1	9.44		O1	1.49
	O2	2.66		O2	9.14		O2	9.38		O2	1.33
	P8	3.05		P8	8.57		P8	3.47		P8	1.47
	T8	2.42		T8	9.43		T8	7.85		T8	1.24
	FC6	2.85		FC6	8.76		FC6	3.41		FC6	1.23
	F4	2.59		F4*	7.05*		F4	7.85		F4	0.93
	F8	2.61		F8	9.75		F8	7.16		F8	0.99

	AF4	2.94		AF4	8.98		AF4	5.48		AF4*	0.85*
30 (#8)	AF3	11.99									
	F7	10.58									
	F3	15.40									
	FC5	15.47									
	T7	14.89									
	P7	10.33									
	O1	14.18									
	O2	12.36									
	P8	9.34									
	T8	13.32									
	FC6	14.04									
	F4	13.92									
	F8	14.67									
	AF4	13.32									

v. Time series filterd into the gamma band (31–55 Hz)

Table S13: Table entries are analogous to Table S9 but for the time series filtered into the gamma band (31–55 Hz).

Sub ID	Sensor	MSE	Sub ID	Sensor	MSE	Sub ID	Sensor	MSE	Sub ID	Sensor	MSE
1 (#3)	AF3	1.79	2 (#5)	AF3	5.96	3 (#6)	AF3	2.58	4 (#5)	AF3	2.40
	F7	1.68		F7	5.04		F7	1.80		F7	1.82
	F3	1.68		F3	5.82		F3	3.28		F3	1.54
	FC5	1.62		FC5	4.76		FC5	2.30		FC5	2.33
	T7	1.73		T7	4.04		T7	2.03		T7	2.06
	P7	1.51		P7	5.02		P7	2.21		P7	1.57
	O1	1.79		O1	4.44		O1	2.77		O1	1.83
	O2	1.68		O2	5.00		O2	4.25		O2	2.56
	P8	1.62		P8	5.60		P8	1.93		P8	2.17
	T8	1.73		T8	5.82		T8*	1.62*		T8	1.51
	FC6	1.96		FC6	5.84		FC6	1.86		FC6	2.58
	F4	1.62		F4	4.78		F4	2.56		F4	1.39
	F8	1.96		F8	3.62		F8	1.79		F8	1.42
	AF4	1.68		AF4	4.48		AF4	1.99		AF4	1.58
5 (#5)	AF3	1.36	6 (#10)	AF3	12.89	7 (#6)	AF3	4.41	8 (#6)	AF3	4.18
	F7	1.55		F7	7.17		F7	2.48		F7	2.75

	F3	1.78		F3	11.32		F3	4.96		F3	3.69
	FC5	1.59		FC5	6.77		FC5	3.61		FC5	1.54
	T7	1.68		T7*	6.73*		T7	2.00		T7	3.90
	P7	1.49		P7	13.12		P7	2.45		P7	5.22
	O1	1.78		O1	15.36		O1	2.38		O1	1.45
	O2	1.27		O2	11.27		O2	5.13		O2	1.82
	P8	2.57		P8	13.44		P8	5.70		P8	1.73
	T8	1.32		T8	14.79		T8	2.71		T8	1.66
	FC6	1.28		FC6	19.01		FC6	3.45		FC6	3.36
	F4	1.36		F4	12.21		F4	5.88		F4	1.66
	F8	1.34		F8	12.38		F8	5.17		F8	3.13
	AF4	1.27		AF4	16.67		AF4	2.19		AF4	2.31
9	AF3	1.14	10	AF3	1.11	11	AF3	0.48	13	AF3	9.84
(#4)	F7	1.11	(#4)	F7	1.20	(#3)	F7	0.51	(#10)	F7	9.63
	F3	2.72		F3	1.14		F3	0.40		F3	10.68
	FC5	1.13		FC5	1.10		FC5	0.45		FC5	11.12
	T7	0.94		T7	0.94		T7	0.40		T7	13.97
	P7	1.07		P7	1.11		P7	0.49		P7	10.21
	O1	1.01		O1	1.02		O1	0.60		O1	8.41
	O2	1.21		O2	1.16		O2	0.55		O2	9.72
	P8	1.17		P8	1.30		P8	0.52		P8	8.74
	T8	1.21		T8	1.19		T8	0.48		T8	10.87
	FC6	1.14		FC6	0.89		FC6	0.51		FC6	10.18
	F4	1.32		F4	0.57		F4	0.47		F4	11.44
	F8	1.18		F8	1.09		F8	0.57		F8	10.28
	AF4	1.10		AF4	1.28		AF4	0.51		AF4	13.65
14	AF3	4.49	15	AF3	27.59	16	AF3	1.24	17	AF3	0.65
(#5)	F7	1.65	(#6)	F7	8.88	(#4)	F7	1.47	(#3)	F7	1.16
	F3	4.93		F3	9.75		F3	2.35		F3	0.84
	FC5	4.09		FC5	11.36		FC5	2.51		FC5	0.89
	T7	2.56		T7	7.57		T7	1.69		T7	0.81
	P7	1.66		P7	7.67		P7	1.22		P7	0.75
	O1	1.54		O1	11.84		O1	1.64		O1	0.69
	O2	1.59		O2	11.54		O2	1.40		O2	0.56
	P8	1.99		P8	6.97		P8	1.33		P8	0.76
	T8	3.06		T8	6.62		T8	1.78		T8	0.92
	FC6	4.09		FC6	6.83		FC6	2.24		FC6	0.73
	F4	2.07		F4	7.32		F4	1.18		F4	0.73
	F8	4.38		F8	11.81		F8	2.20		F8	1.13
	AF4	4.38		AF4	27.87		AF4	0.95		AF4	0.71
18	AF3	5.33	19	AF3	3.55	20	AF3	4.46	21	AF3	6.53
(#6)	F7	3.91	(#5)	F7	6.30	(#6)	F7	4.10	(#6)	F7	7.68

	F3	3.03		F3	4.96		F3	1.78		F3	2.66
	FC5	5.64		FC5	5.06		FC5	4.56		FC5	3.88
	T7	6.13		T7	7.44		T7	1.97		T7	2.19
	P7	5.78		P7	4.89		P7	6.22		P7	3.91
	O1	5.66		O1	4.51		O1	5.23		O1	4.39
	O2	4.87		O2	4.83		O2	2.63		O2	2.69
	P8	7.70		P8	5.70		P8	2.76		P8	1.74
	T8	6.09		T8	5.85		T8	3.84		T8	2.57
	FC6	4.93		FC6	5.75		FC6	3.84		FC6	3.61
	F4	6.76		F4	4.17		F4	3.75		F4	2.75
	F8	11.99		F8	5.39		F8	4.39		F8	3.00
	AF4	10.31		AF4	3.55		AF4	1.78		AF4	3.11
22	AF3	7.27	23	AF3	1.90	24	AF3	3.37	25	AF3	2.82
(#7)	F7	6.52	(#4)	F7	1.98	(#7)	F7	5.50	(#6)	F7	6.47
	F3	5.75		F3	1.91		F3	3.86		F3	3.88
	FC5	9.36		FC5	1.91		FC5	3.83		FC5	4.63
	T7	4.45		T7	1.88		T7	5.51		T7	2.81
	P7	4.82		P7	1.99		P7	5.64		P7	2.92
	O1*	2.61*		O1	2.72		O1	8.70		O1	4.59
	O2	3.06		O2	2.20		O2	5.79		O2	3.42
	P8	3.03		P8	2.33		P8	4.59		P8	4.18
	T8	9.56		T8	2.43		T8	3.18		T8	3.51
	FC6	4.69		FC6	2.24		FC6	4.13		FC6	3.78
	F4	3.64		F4	2.12		F4*	3.13*		F4	3.29
	F8	7.10		F8	2.25		F8	3.89		F8	3.68
	AF4	5.97		AF4	1.86		AF4	3.74		AF4	3.78
26	AF3	2.51	27	AF3	7.60	28	AF3	6.31	29	AF3	1.56
(#6)	F7	2.74	(#7)	F7	10.15	(#6)	F7	7.86	(#3)	F7	1.47
	F3	3.01		F3	9.57		F3	8.48		F3	1.13
	FC5	3.16		FC5	10.21		FC5	7.94		FC5	1.59
	T7	3.19		T7	8.72		T7	3.88		T7	2.04
	P7	2.61		P7	9.40		P7	8.44		P7	1.40
	O1	3.02		O1	11.36		O1	9.65		O1	1.47
	O2	2.96		O2	9.86		O2	8.96		O2	1.31
	P8	3.81		P8	8.49		P8	3.27		P8	1.37
	T8	2.81		T8	9.56		T8	8.65		T8	1.25
	FC6	2.76		FC6	10.40		FC6	3.63		FC6	1.27
	F4	2.24		F4	11.40		F4	7.73		F4	1.08
	F8	2.34		F8	9.34		F8	7.91		F8	1.16
	AF4	2.99		AF4	9.86		AF4	7.95		AF4	1.13
30	AF3	14.25									
(#8)	F7	8.73									

	F3	10.19									
	FC5	10.74									
	T7	14.18									
	P7	8.73									
	O1	10.68									
	O2	14.21									
	P8	12.55									
	T8	11.36									
	FC6	15.30									
	F4	14.74									
	F8	13.15									
	AF4	13.29									

vi. Time series not filtered into any band

Table S14: Table entries are analogous to Table S9 but for time series not filtered into any frequency band.

Sub ID	Sensor	MSE	Sub ID	Sensor	MSE	Sub ID	Sensor	MSE	Sub ID	Sensor	MSE
1	AF3	1.56	2	AF3	5.84	3	AF3	2.46	4	AF3	2.71
(#3)	F7	1.79	(#5)	F7	5.20	(#6)	F7	2.73	(#5)	F7	2.65
	F3	2.01		F3	5.20		F3	2.27		F3	2.08
	FC5	1.85		FC5	5.34		FC5	2.90		FC5	2.61
	T7	2.25		T7	4.78		T7	2.27		T7	2.56
	P7	1.51		P7	6.00		P7	2.45		P7	1.69
	O1	1.85		O1	5.26		O1	2.68		O1	1.93
	O2	1.32		O2	5.12		O2	2.92		O2	2.25
	P8	1.51		P8	5.78		P8	2.94		P8	2.40
	T8	1.68		T8	4.88		T8	1.89		T8	1.76
	FC6	1.51		FC6*	2.62*		FC6	2.41		FC6	1.67
	F4	1.85		F4	4.96		F4	2.35		F4	1.58
	F8	2.30		F8	3.70		F8	2.10		F8	1.57
	AF4	1.51		AF4	3.08		AF4	2.56		AF4	1.68
5	AF3	1.16	6	AF3	16.31	7	AF3	3.12	8	AF3	2.90
(#5)	F7	1.19	(#10)	F7	13.11	(#6)	F7	2.26	(#6)	F7	2.69
	F3	1.59		F3	16.74		F3	5.09		F3	2.70
	FC5	1.41		FC5	10.54		FC5	2.30		FC5	1.46
	T7	1.35		T7	6.86		T7	2.28		T7	2.39
	P7	1.09		P7	14.49		P7	2.58		P7	4.67

	O1	1.53		O1	12.6		O1	3.33		O1	1.45
	O2	1.15		O2	10.59		O2	2.52		O2	1.72
	P8	3.51		P8	16.79		P8	2.54		P8	1.30
	T8	1.43		T8	10.31		T8	2.91		T8	1.66
	FC6	1.58		FC6	17.73		FC6	2.81		FC6	4.24
	F4	1.41		F4	14.14		F4	4.39		F4	2.01
	F8	1.43		F8	12.65		F8	4.97		F8	2.13
	AF4	1.30		AF4	15.75		AF4	2.33		AF4	1.63
9	AF3	1.11	10	AF3	0.85	11	AF3	0.43	13	AF3	11.31
(#4)	F7	1.11	(#4)	F7	1.07	(#3)	F7	0.32	(#10)	F7	10.04
	F3	2.94		F3	0.94		F3	0.36		F3	11.16
	FC5	1.59		FC5	1.25		FC5*	0.31*		FC5	10.00
	T7	0.96		T7	0.88		T7*	0.31*		T7	10.71
	P7	0.97		P7	1.23		P7	0.32		P7	12.06
	O1	0.96		O1	1.17		O1	0.33		O1	12.12
	O2	1.35		O2	0.65		O2	0.37		O2	9.12
	P8	1.27		P8	1.05		P8	0.37		P8	9.22
	T8	1.46		T8	0.94		T8	0.35		T8	10.66
	FC6	1.52		FC6	0.58		FC6	0.44		FC6	11.01
	F4	1.18		F4*	0.47*		F4	0.48		F4	11.99
	F8	1.17		F8	0.64		F8	0.52		F8	12.38
	AF4	1.11		AF4	0.53		AF4	0.44		AF4	14.16
14	AF3	3.81	15	AF3	27.16	16	AF3	0.95	17	AF3	0.64
(#5)	F7	1.72	(#6)	F7	6.62	(#4)	F7	0.98	(#3)	F7	1.12
	F3	5.65		F3	6.62		F3	1.11		F3	0.61
	FC5	4.62		FC5	8.90		FC5	2.60		FC5	0.68
	T7	2.63		T7	8.62		T7	1.82		T7	1.51
	P7	3.38		P7	6.62		P7	1.29		P7	0.60
	O1	1.56		O1	12.7		O1	3.09		O1	0.55
	O2	1.63		O2	9.72		O2	2.51		O2	0.51
	P8	1.76		P8	8.26		P8	1.29		P8	0.57
	T8	2.97		T8	6.62		T8	2.22		T8	0.69
	FC6	4.47		FC6	6.39		FC6	2.07		FC6	0.55
	F4	1.57		F4	6.62		F4	1.15		F4	0.71
	F8	2.81		F8	13.17		F8	1.00		F8*	0.49*
	AF4	3.84		AF4	25.26		AF4	0.95		AF4	0.60
18	AF3	3.21	19	AF3	7.01	20	AF3	3.29	21	AF3	3.40
(#6)	F7	3.61	(#5)	F7	4.14	(#6)	F7	3.62	(#6)	F7	7.52
	F3	3.10		F3	6.92		F3	1.76		F3	2.31
	FC5	3.72		FC5	5.45		FC5	4.27		FC5	2.35
	T7	5.18		T7	7.65		T7	1.94		T7	1.83
	P7	5.55		P7	7.14		P7	6.41		P7	4.19

	O1	5.06		O1	5.68		O1	4.30		O1	5.03
	O2	5.94		O2	3.55		O2	4.40		O2	2.47
	P8	3.99		P8	4.20		P8	4.42		P8	1.68
	T8	4.84		T8	8.59		T8	3.44		T8	2.76
	FC6	3.21		FC6	3.97		FC6	3.65		FC6	2.23
	F4	5.75		F4	7.77		F4	4.05		F4	2.51
	F8	5.55		F8	5.77		F8	5.65		F8	3.70
	AF4	5.37		AF4	7.73		AF4	2.43		AF4	2.09
22	AF3	4.21	23	AF3	1.92	24	AF3	5.86	25	AF3	3.08
(#7)	F7	5.55	(#4)	F7	2.14	(#7)	F7	5.08	(#6)	F7	6.51
	F3	5.05		F3	2.10		F3	4.46		F3	3.51
	FC5	5.12		FC5	2.09		FC5	6.32		FC5	6.30
	T7	4.88		T7	2.17		T7	5.67		T7	3.59
	P7	10.06		P7	1.91		P7	3.82		P7	3.22
	O1	3.09		O1	3.16		O1	7.50		O1	4.59
	O2	3.19		O2	2.48		O2	5.67		O2	2.62
	P8	4.14		P8	2.45		P8	5.18		P8*	2.38*
	T8	4.51		T8	2.25		T8	4.47		T8	2.60
	FC6	3.17		FC6	1.97		FC6	4.33		FC6	2.96
	F4	4.3		F4	1.78		F4	4.37		F4	3.62
	F8	3.25		F8	2.14		F8	3.66		F8	3.25
	AF4	3.74		AF4	2.14		AF4	4.55		AF4	3.45
26	AF3	2.42	27	AF3	9.93	28	AF3	6.56	29	AF3	1.45
(#6)	F7	2.72	(#7)	F7	9.48	(#6)	F7	7.74	(#3)	F7	1.55
	F3	3.10		F3	8.74		F3	7.79		F3	2.16
	FC5	3.18		FC5	8.57		FC5	7.53		FC5	1.59
	T7	3.10		T7	8.80		T7	3.73		T7	2.12
	P7	2.66		P7	8.89		P7	7.46		P7	1.57
	O1	2.71		O1	8.45		O1	9.11		O1	1.59
	O2	2.42		O2	10.74		O2	8.15		O2	1.20
	P8	3.51		P8	7.61		P8*	3.25*		P8	1.53
	T8	2.71		T8	10.81		T8	7.54		T8	1.77
	FC6	2.62		FC6	9.28		FC6	3.48		FC6	1.47
	F4	2.38		F4	10.74		F4	8.14		F4	0.92
	F8	2.33		F8	9.55		F8	6.21		F8	1.12
	AF4	3.00		AF4	9.60		AF4	4.65		AF4*	0.85*
30	AF3	11.71									
(#8)	F7	6.36									
	F3	11.99									
	FC5	11.86									
	T7	15.01									
	P7	9.66									

	O1	14.96									
	O2	12.30									
	P8	5.51									
	T8	13.96									
	FC6	14.04									
	F4	12.23									
	F8	12.77									
	AF4	9.66									

b. Time series and k-nearest neighbors: Input 2

Table S15: MSE for k-NN (k=1), the input of the time series filtered into a specific frequency band (or not filtered) at all 14 sensors. The first column gives the specific frequency band; the second, fourth, and sixth column give the Subject ID; and the third, fifth, and seventh columns are the calculated MSE. Light-gray-marked values are the lowest MSE for each participant across all frequency bands. Participants with Sub IDs 10 and 22 are left-handed; the rest are right-handed.

Band	Sub ID	MSE	Sub ID	MSE	Sub ID	MSE
Non	1 (#3)	1.90	11 (#3)	0.32	21 (#6)	2.91
0–3		1.93		0.56		2.80
4–7		2.06		0.49		3.20
8–13		1.31		0.68		2.66
14–30		1.79		0.45		2.76
31–55		1.79		0.45		2.75
Non	2(#5)	5.00	12 (#1)	0	22 (#7)	4.35
0–3		6.02		0		3.38
4–7		4.94		0		4.36
8–13		3.18		0		3.83
14–30		5.28		0		4.99
31–55		6.50		0		2.87
Non	3 (#6)	2.58	13 (#10)	9.62	23 (#4)	2.17
0–3		2.59		15.72		2.11
4–7		2.79		11.60		2.40
8–13		2.04		13.03		2.42
14–30		2.89		9.93		2.05
31–55		2.10		8.99		2.51
Non	4 (#5)	1.76	14 (#5)	1.56	24 (#7)	5.11
0–3		1.33		2.13		5.47
4–7		2.88		2.16		4.91

8–13		2.57		1.71		4.88
14–30		1.85		1.56		3.40
31–55		1.60		1.56		4.34
Non	5 (#5)	1.28	15 (#6)	16.88	25 (#6)	2.67
0–3		1.45		15.53		4.60
4–7		1.34		14.07		3.44
8–13		1.32		17.34		2.85
14–30		1.30		13.97		3.52
31–55		1.20		12.87		3.56
Non	6 (#10)	15.90	16 (#4)	1.87	26 (#6)	2.75
0–3		12.53		1.95		2.31
4–7		10.80		1.05		2.76
8–13		15.10		0.85		3.05
14–30		17.02		2.04		2.59
31–55		17.11		1.98		2.63
Non	7 (#6)	2.26	17 (#3)	0.59	27 (#7)	9.11
0–3		2.61		0.77		9.86
4–7		3.25		0.79		9.26
8–13		3.28		0.84		10.00
14–30		2.90		0.64		9.69
31–55		3.26		0.88		11.16
Non	8 (#6)	1.28	18 (#6)	5.48	28 (#6)	8.79
0–3		1.75		3.67		6.96
4–7		2.31		3.61		7.91
8–13		2.93		4.60		7.98
14–30		1.75		4.97		8.93
31–55		1.64		6.16		9.56
Non	9 (#4)	1.07	19 (#5)	7.06	29 (#3)	1.16
0–3		1.03		7.22		1.17
4–7		1.40		5.39		1.79
8–13		0.93		7.49		1.91
14–30		1.15		4.55		0.97
31–55		1.15		3.69		1.25
Non	10 (#4)	0.78	20 (#6)	2.82	30 (#8)	14.16
0–3		0.74		2.88		6.66
4–7		0.59		2.48		8.43
8–13		0.90		2.33		8.03
14–30		1.30		3.47		14.92
31–55		1.20		3.39		13.85

c. Time series and k-nearest neighbors: Input 3

Table S16: MSE for k-NN (k=1) using the time series filtered into all five frequency bands at all 14 sensors as input. The first column in each subtable gives the Subject ID, followed by the calculated MSE and the number of different SWB values given by the participant. Participants with Sub IDs 10 and 22 are left-handed; the rest are right-handed.

Sub ID	MSE	#SWB	Sub ID	MSE	#SWB	Sub ID	MSE	#SWB	Sub ID	MSE	#SWB
1	1.79	3	9	1.12	4	18	14.93	6	26	2.56	6
2	4.48	4	10	1.36	4	19	6.30	5	27	9.19	7
3	3.14	6	11	0.51	3	20	3.18	6	28	8.23	6
4	2.19	5	13	8.29	10	21	2.01	6	29	1.28	3
5	1.35	5	14	4.79	5	22	5.52	7	30	5.96	8
6	13.15	10	15	6.97	6	23	1.27	4			
7	3.26	6	16	1.55	4	24	7.01	7			
8	2.24	6	17	0.83	3	25	5.64	6			

Table S17: MSE for k-NN (k=1) using the time series filtered into all five frequency bands and the unfiltered time series at all 14 sensors as input. The first column in each subtable gives the Subject ID, followed by the calculated MSE and the number of different SWB values given by the participant. Participants with Sub IDs 10 and 22 are left-handed; the rest are right-handed.

Sub ID	MSE	#SWB	Sub ID	MSE	#SWB	Sub ID	MSE	#SWB	Sub ID	MSE	#SWB
1	2.01	3	9	0.94	4	18	5.48	6	26	2.74	6
2	5.78	4	10	0.77	4	19	7.10	5	27	8.90	7
3	2.48	6	11	0.33	3	20	2.76	6	28	8.79	6
4	1.46	5	13	9.40	10	21	2.83	6	29	1.24	3
5	1.22	5	14	1.56	5	22	4.57	7	30	13.31	8
6	16.43	10	15	16.51	6	23	2.15	4			
7	2.16	6	16	1.67	4	24	5.08	7			
8	1.31	6	17	0.60	3	25	2.70	6			

4. Clustering:

a. FAA

Table S18: Label for each participant based on the distance matrix from the FAA algorithm and after Isomap embedding. The first column gives the Subject ID. k indicates the number of clusters.

The entries indicate the cluster number to which each participant belongs. SC gives the Silhouette coefficient for that clustering. Participants with Sub IDs 10 and 22 are left-handed; the rest are right-handed.

Sub_ID	K=2	K=3	K=4	K=5	K=6
1	1	1	3	3	3
2	1	1	3	3	3
3	1	1	1	4	4
4	0	0	0	0	0
5	1	2	2	2	2
6	1	1	1	4	4
7	1	1	1	1	1
8	0	0	0	0	0
9	1	2	2	2	2
10	1	2	2	2	2
11	1	1	1	1	1
13	1	1	1	1	1
14	0	0	0	0	0
15	0	0	1	1	1
16	0	0	0	0	0
17	0	0	0	0	0
18	1	1	1	4	4
19	1	1	1	1	1
20	0	2	2	2	5
21	1	1	1	1	1
22	1	1	1	1	1
23	1	1	1	4	4
24	1	1	3	3	3
25	0	0	0	0	0
26	1	2	3	3	5
27	1	1	3	3	3
28	1	1	3	3	3
30	1	1	1	1	1
SC	0.429	0.480	0.561	0.648	0.666

b. Relative Power k-NN

Table S19: Label for each participant using the distance matrix from the relative power algorithm and after Isomap embedding. The first column gives the subject ID. k indicates the number of clusters. The entries indicate the cluster number to which each participant belongs. SC gives the

Silhouette coefficient for that clustering. Participants with Sub IDs 10 and 22 are left-handed; the rest are right-handed.

Sub_ID	K=2	K=3	K=4	K=5	K=6
1	1	2	2	2	2
2	1	2	3	3	3
3	0	1	1	1	1
4	0	0	0	0	0
5	1	2	2	2	2
6	0	0	1	4	4
7	0	1	1	1	1
8	1	1	1	4	5
9	0	0	0	0	0
10	1	2	2	2	2
11	1	2	3	3	3
13	0	1	1	1	1
14	1	2	2	2	2
15	0	0	0	0	0
16	0	1	1	4	4
17	0	0	0	0	0
18	0	1	1	1	1
19	0	0	0	0	0
20	0	0	0	0	0
21	1	1	3	3	5
22	0	0	1	4	4
23	0	1	1	4	4
24	0	1	1	4	4
25	0	0	1	4	4
26	0	0	0	0	0
27	1	1	3	3	5
28	0	0	0	0	0
30	0	0	0	0	0
SC	0.354	0.403	0.423	0.410	0.399

c. Relative power linear regression

Table S20: Label for each participant when using the distance matrix from the relative power linear regression algorithm and after Isomap embedding. The first column gives the subject ID. k indicates the number of clusters. The entries indicate the cluster number to which each participant belongs. SC gives the Silhouette coefficient for that clustering. Participants with Sub IDs 10 and 22 are left-handed; the rest are right-handed.

Sub_ID	K=2	K=3	K=4	K=5	K=6
1	0	0	0	0	0
2	1	2	2	2	5
3	1	1	1	1	1
4	0	0	0	4	4
5	1	2	2	2	5
6	1	1	1	1	1
7	1	2	2	2	2
8	0	0	0	0	0
9	0	0	3	0	0
10	1	2	2	2	2
11	1	2	2	2	2
13	1	1	1	1	1
14	0	0	0	0	0
15	1	1	1	1	1
16	0	2	3	3	3
17	0	0	0	0	0
18	1	1	1	1	1
19	1	1	1	1	1
20	0	0	0	0	0
21	1	1	1	1	1
22	1	1	1	1	1
23	1	1	1	1	1
24	1	2	2	2	2
25	1	2	2	2	5
26	0	2	3	3	3
27	0	0	0	4	4
28	0	0	0	4	4
30	1	1	1	1	1
SC	0.441	0.548	0.562	0.524	0.512

d. Times Series

Table S21: Label for each participant when using the distance matrix from the time series algorithm and after Isomap embedding. The first column gives the subject ID. k indicates the number of clusters. The entries indicate the cluster number to which each participant belongs. SC gives the Silhouette coefficient for that clustering. Participants with Sub IDs 10 and 22 are left-handed; the rest are right-handed.

Sub_ID	K=2	K=3	K=4	K=5	K=6
1	1	2	1	2	1

2	1	1	3	3	3
3	1	2	1	2	1
4	0	0	2	4	5
5	1	2	1	2	2
6	1	2	1	2	2
7	1	1	3	3	3
8	0	0	2	4	5
9	1	2	1	2	2
10	1	2	1	2	2
11	1	1	3	3	3
13	0	0	0	0	0
14	0	0	2	4	5
15	0	0	0	0	0
16	0	0	2	4	5
17	0	0	0	0	0
18	1	2	1	1	1
19	0	0	2	4	5
20	0	0	2	4	4
21	1	2	1	2	2
22	1	2	1	1	1
23	1	2	1	2	2
24	1	2	1	2	2
25	0	0	0	0	0
26	0	0	2	4	5
27	1	1	3	1	1
28	1	2	1	2	2
30	1	1	3	3	3
SC	0.443	0.560	0.614	0.522	0.427
